# Pharmacogenetic Evidence in Opioid-Related Toxicity and Death with an Appraisal of Emerging Multi-Omics Studies: A Systematic Review

**DOI:** 10.3390/ijms27188332

**Published:** 2026-09-19

**Authors:** Tommaso D’Anna, Angelo Montana, Giulia La Magna, Marco Piraino, Antonella Pecorella, Maria Giovanna Scioli, Sonia Terriaca, Beatrice Belmonte, Augusto Orlandi, Stefania Zerbo, Francesco Paolo Busardò, Antonina Argo

**Affiliations:** 1 Department of Biomedicine and Prevention, Tor Vergata University of Rome, 00133 Rome, Italy; scioli@med.uniroma2.it (M.G.S.); terriacasonia093@gmail.com (S.T.); orlandi@uniroma2.it (A.O.); 2 Tumor Immunology Unit, Department of Health Promotion, Mother and Child Care, Internal Medicine and Medical Specialties (PROMISE), University of Palermo, 90127 Palermo, Italy; beatrice.belmonte@unipa.it; 3 Legal Medicine Unit, University Hospital “Paolo Giaccone”, 90127 Palermo, Italy; stefania.zerbo@unipa.it (S.Z.); antonella.argo@unipa.it (A.A.); 4 Section of Legal Medicine, Department of Biomedical Sciences and Public Health, Marche Polytechnic University, 60126 Ancona, Italy; f.p.busardo@staff.univpm.it; 5 Department of Health Promotion, Mother and Child Care, Internal Medicine and Medical Specialties (PROMISE), University of Palermo, 90127 Palermo, Italy; giulia.la.magna.glm@gmail.com (G.L.M.); mpiraino152@gmail.com (M.P.);; 6 Research Center of Legal Medicine and Risk Management, Cardiovascular Department, Maria Cecilia Hospital, GVM Care & Research, 48033 Cotignola, Italy

**Keywords:** pharmacogenetics, pharmacogenomics, multi-omics, opioids, forensic toxicology, postmortem toxicology, molecular toxicology, cause of death

## Abstract

Interpretation of opioid-related toxicity and death requires integration of exposure, measured toxicology, individual susceptibility, and alternative causes. We systematically reviewed human pharmacogenetic/pharmacogenomic (PGx) and multi-omic studies published from 1 January 2008 to March 2026, identified through MEDLINE/PubMed, Scopus, and Web of Science Core Collection. JBI tools informed risk-of-bias assessment, and findings underwent effect-size-oriented narrative synthesis and descriptive mapping. The 75-study corpus was predominantly PGx (51 studies, 68.0%); only 17 studies addressed transcriptomic/epigenetic, metabolomic, proteomic, or integrated approaches, and seven retained other molecular classifications. These domains therefore differ substantially in evidential maturity. The most coherent PGx findings concerned CYP2D6-dependent codeine and tramadol bioactivation, CYP2B6-dependent methadone disposition, and ABCB1-related tissue distribution. Omics findings were exploratory, with limited external validation and specificity for fatal causation; proteomics rested on a single investigation. Forensic interpretative directness was high or moderate in 36 studies and low or absent in 39. No nitazene-specific study met this review’s molecular eligibility criteria. Certainty was very low for the four outcome-focused bodies assessed with GRADE; integration utility and confounder control were appraised narratively. Molecular findings may explain discordance between inherited susceptibility, parent-drug/metabolite patterns, and the observed phenotype. Downstream signatures may also reflect chronic exposure, terminal hypoxia, or postmortem change. Neither the descriptive evidence-map scores nor individual molecular markers provide validated estimates of forensic risk or establish cause of death independently.

## 1. Introduction

Opioid-related morbidity and mortality have become a major international public health and forensic challenge. During the past two decades, the epidemiology of opioid-related death has shifted from prescription opioids and heroin toward increasingly potent synthetic opioids, particularly illicitly manufactured fentanyl and its analogues in North America, while European drug markets remain heterogeneous and increasingly include pharmaceutical opioids, opioid agonist medications, fentanyl derivatives, nitazenes, and other novel synthetic opioids [1,2,3,4,5]. Fatal cases also frequently involve multiple substances, including benzodiazepines, stimulants, gabapentinoids, alcohol, and xylazine [3,4,5,6,7,8,9]. These changing exposure patterns have made causal interpretation progressively more complex and have reinforced a fundamental principle of forensic toxicology: the presence or concentration of an opioid cannot, in isolation, establish that the drug caused death [6,10,11].

Forensic interpretation is particularly complex because therapeutic, toxic, and fatal opioid concentrations overlap extensively [6,10,11]. Measured values are influenced by dose, route and timing of administration, survival interval, tolerance, renal and hepatic function, co-intoxicants, specimen type, residual metabolism, postmortem redistribution, and compartment-specific drug distribution [6,10,11]. Highly lipophilic opioids such as fentanyl rapidly penetrate the central nervous system (CNS), and measured postmortem concentrations depend on anatomical sampling site and tissue distribution [10,12]. Opioid lethality is primarily mediated by μ-opioid-receptor-dependent depression of respiratory rhythm generation and ventilatory responses to hypercapnia and hypoxemia, although κ- and δ-receptors and suprapontine pathways also contribute to respiratory control [13,14,15]. Consequently, detection of an opioid or comparison with a conventional lethal range cannot independently establish causation, particularly in cases involving borderline concentrations, chronic tolerance, prolonged survival, or several respiratory depressants [6,10,11,13,14,15]. Concentration-based interpretation must be integrated with the circumstances of exposure, toxicological profile, autopsy and histopathological findings, comorbidity, tolerance, and plausible alternative causes of death.

A substantial component of interindividual variability is genetically mediated. Pharmacogenetic and pharmacogenomic (PGx) variation affects opioid bioactivation, clearance, glucuronidation, membrane transport, receptor signaling, dose requirement, and adverse effects [16,17,18]. CYP2D6 converts codeine and tramadol into morphine and O-desmethyltramadol and contributes to hydrocodone and oxycodone metabolism; functional alleles, gene deletions, duplications, and copy-number variation generate poor, intermediate, normal, and ultrarapid metabolizer phenotypes [16,17,18,19]. CYP2B6 has a stereoselective role in methadone clearance, whereas CYP3A4/5 participates in the disposition of fentanyl, sufentanil, methadone, oxycodone, and other opioids [12,16,17,18]. UGT2B7-mediated glucuronidation regulates the formation of morphine-3-glucuronide and morphine-6-glucuronide, while ABCB1-encoded P-glycoprotein modulates opioid efflux across biological barriers and may influence CNS and target-tissue exposure [16,17,18]. Pharmacodynamic (PD) variability additionally involves OPRM1, COMT, and other genes regulating receptor expression, β-arrestin signaling, catecholaminergic tone, nociception, ventilatory response, and adverse effects [16,17,18,19]. The clinical and forensic consistency of pharmacodynamic candidate-gene associations is, however, substantially more variable than that of the best-supported metabolic pathways.

Genotype represents inherited functional potential rather than the metabolic state operating at the time of intoxication. Enzyme inhibition or induction, competing substrates, drug–drug–gene interactions, inflammation, ancestry, age, pregnancy, chronic exposure, and renal or hepatic dysfunction may modify the expected phenotype and produce phenoconversion [16,17,18,19]. This distinction is critical in forensic toxicology. A genetically predicted metabolizer phenotype becomes evidentially meaningful only when evaluated against the realized toxicological phenotype, including parent-drug and metabolite concentrations, metabolic ratios, tissue distribution, co-exposures, organ function, and the temporal circumstances of exposure. Pharmacogenetic information may therefore strengthen biological plausibility or explain an atypical toxicological pattern, but it should not be interpreted as an autonomous molecular cause of death.

PGx characterizes inherited susceptibility, whereas high-throughput omics technologies interrogate downstream molecular responses to acute intoxication, chronic exposure, tolerance, opioid use disorder (OUD), hypoxia, and terminal injury. Genomic, transcriptomic, and epigenomic studies have implicated OPRM1, FURIN, the SCAI/PPP6C/RABEPK region, GABAergic and glutamatergic neurotransmission, GPCR and Trk signaling, MAPK/ERK regulation, synaptic plasticity, and neuroimmune pathways [20,21,22]. DNA methylation, hydroxymethylation, histone acetylation, chromatin accessibility, miRNAs, and long non-coding RNAs may mediate persistent transcriptional adaptations following opioid exposure [20,21,22]. Metabolomic, lipidomic, and proteomic approaches additionally capture mitochondrial dysfunction, altered fatty-acid oxidation, oxidative stress, membrane remodeling, extracellular matrix (ECM) changes, inflammatory signaling, and neuronal injury [21,22,23].

These molecular layers are complementary but not interchangeable. Germline variation represents pre-existing susceptibility, conventional toxicology characterizes documented exposure and realized metabolism, and downstream omics signatures reflect biological response [12,16,17,18,19,20,21,22,23]. A transcriptomic, epigenetic, or metabolomic profile may discriminate exposed from unexposed individuals while remaining non-specific for fatal causation if it predominantly reflects chronic OUD, neuroadaptation, terminal hypoxia, or generalized agonal stress [20,21,22,23]. Likewise, high apparent discrimination in a derivation dataset does not establish external validity, molecular specificity, or applicability to individual cause-of-death assessment. Evidentiary value therefore depends on precision, multiplicity control, replication, biological matrix, temporal relevance, mechanistic plausibility, and coherence with conventional toxicology, scene investigation, autopsy, histopathology, postmortem conditions, and alternative causes of death [6,10,20,21,22,23]. In concise terms, genotype addresses susceptibility, conventional toxicology characterizes exposure and realized pharmacokinetics, and downstream omics characterize biological response.

The rapid emergence of fentanyl analogues and benzimidazole opioids exposes a mismatch between contemporary forensic casework and the available molecular evidence [4,5,12,24]. Highly potent synthetic opioids present particular interpretative challenges because of rapid central nervous system penetration, low active concentrations, polysubstance involvement, evolving analytical targets, and limited substance-specific interpretative data [12,24]. Despite their current forensic importance, validated PGx or multi-omic biomarkers capable of informing fatal intoxication by fentanyl analogues or nitazenes remain poorly developed.

Previous reviews have examined individual components of this field, including pharmacogenetics of lethal opioid overdose, precision prescribing, OPRM1-related effects, functional genomics of opioid action and opioid use disorder, and the pharmacology and analytical toxicology of fentanyl and novel synthetic opioids [12,16,17,18,24,25,26]. However, these domains have largely remained separated. A clinically derived genetic association does not automatically translate into postmortem causal evidence, and a downstream molecular signature does not necessarily identify the toxicant responsible for death. An integrated assessment therefore requires explicit separation of inherited susceptibility, realized pharmacokinetic/pharmacodynamic phenotype, and downstream molecular response.

Accordingly, this systematic review aimed to integrate PGx and multi-omic evidence published from 2008 to March 2026 and relevant to opioid exposure, toxicity, overdose, and death within a forensic interpretative framework. Specifically, we sought to (i) identify genetic variants, metabolic phenotypes, molecular signatures, and biological pathways associated with opioid pharmacokinetics, pharmacodynamics, adverse effects, and fatal outcomes; (ii) compare findings across opioid classes, exposure contexts, biological matrices, and molecular platforms and evaluate their magnitude, precision, multiplicity control, validation, reproducibility, and forensic directness; and (iii) determine where molecular information can meaningfully explain discordance between documented exposure, measured concentrations, individual susceptibility, and the observed toxic or fatal phenotype. We hypothesized that the greatest forensic value would arise not from any single genetic or omic marker, but from evidentiary convergence across inherited susceptibility, realized toxicological phenotype, downstream biological response, and the complete medicolegal investigation.

## 2. Methods

### 2.1. Study Design, Methodological Framework, and Registration

This systematic review was conducted and reported in accordance with PRISMA 2020 [27]. The protocol was developed with reference to PRISMA-P and JBI guidance for reviews of etiology and association [28,29,30]. The protocol and review materials were deposited on the Open Science Framework (OSF; https://osf.io/j2gsy, accessed on 27 June 2026). The OSF record was created after completion of full-text study selection and before data extraction; it should therefore be regarded as a publicly archived protocol rather than a prospective preregistration. Any differences between the archived protocol and the final review procedures are reported in the OSF materials and/or Supplementary Material. No changes to eligibility were made after data extraction commenced.

### 2.2. Eligibility Criteria

Eligibility criteria were defined using the Population, Exposure, Outcome, and Study design (PEOS) framework recommended for reviews of association [29,30]. Eligible populations included living or deceased human subjects in therapeutic, clinical, non-medical, toxicological, or autopsy settings, as well as human biological specimens obtained from these subjects. Eligible exposures comprised licit and illicit opioids, including natural, semisynthetic, and synthetic compounds, fentanyl and its analogues, benzimidazole opioids such as nitazenes, heroin, methadone, buprenorphine, tramadol, codeine, morphine, oxycodone, hydrocodone, and mixed-opioid exposures. Outcomes of interest included PGx variants affecting opioid PK/PD, toxicity, or fatal outcome, together with metabolomic, lipidomic, proteomic, transcriptomic, epigenetic, methylomic, microbiomic, and integrated multi-omic biomarkers associated with opioid exposure, adverse effects, overdose, or death. Original observational, experimental, and translational studies, including cohort, case–control, cross-sectional, case-series, and case-report designs, were eligible. Study protocols, reviews, editorials, commentaries, letters without original data, conference proceedings, dissertations, theses, and other non-peer-reviewed or grey-literature sources were excluded. Only English-language studies published from 1 January 2008 onwards were eligible.

The lower publication-date limit of 1 January 2008 was specified in the archived protocol in addition to the PEOS criteria. Publication years are a distinct report-level eligibility characteristic [28]. The common 2008–March 2026 window defines a contemporary cross-domain synthesis of PGx and emerging omics evidence, informed by the expansion of high-throughput profiling around this period, including quantitative mammalian RNA sequencing and base-resolution human methylome mapping [31,32]. These milestones do not mark the origin of opioid PGx or establish 2008 as a biological threshold. Foundational codeine studies by Sindrup et al. (1990), Gasche et al. (2004), and Kirchheiner et al. (2007) remain relevant historical context [33,34,35], but are outside the formal publication window. To quantify the earlier evidence recovered by our search strategy, a supplementary pre-2008 eligibility audit was undertaken during peer review and is reported separately (Section 2.3 and Section 3.1; Supplementary Table S5). This audit did not extend the formal synthesis period or add historical reports to the 75-study corpus, its risk-of-bias or certainty appraisals, or its descriptive scores.

### 2.3. Information Sources, Search Strategy, and Study Selection

A systematic search was conducted in MEDLINE/PubMed, Scopus, and Web of Science Core Collection. An initial limited PubMed search was used to identify relevant text words and indexing terms, which informed the complete database-specific strategies. The database search was completed in March 2026; the publication-date eligibility window began on 1 January 2008.

The search combined three concept blocks with AND: opioid exposure; PGx, genomic, or multi-omic biomarkers; and toxicity, overdose, postmortem, cause-of-death, or forensic context. Terms within each block were combined with OR. The opioid block explicitly contained nitazene, isotonitazene, and benzimidazole: these appear as [tiab] terms in PubMed, within TITLE-ABS-KEY in Scopus, and as Topic terms in Web of Science. Complete database-specific strings are reproduced in Supplementary Table S1, rows 1–3. Drug detection, exogenous metabolite identification, or receptor pharmacology alone did not constitute a human PGx or endogenous omics outcome.

Clinical trial registries, patent databases, unpublished studies, non-peer-reviewed search engines, and other grey-literature sources were not searched. Retrieved references were imported into Rayyan, where duplicates were removed and the records were screened against the prespecified eligibility criteria [36]. Following calibration, two reviewers independently screened titles and abstracts and assessed potentially eligible full texts. Full-text exclusion reasons were recorded, and disagreements were resolved through discussion. Reference lists of included studies were also examined for additional records, and the selection process was reported in a PRISMA 2020 flow diagram [27].

On 14 September 2026, a supplementary historical search applied the original strategies in MEDLINE/PubMed, Scopus, and Web of Science Core Collection to publications dated before 1 January 2008, retaining their three-block structure and search terms (Supplementary Table S1). After deduplication, T.D. and A.M. independently screened titles and abstracts and assessed potentially relevant reports in full text using the same selection procedures as the formal review, resolving disagreements through discussion. Assessment used the non-temporal PEOS criteria. The molecular criterion required an opioid-related analysis of genetic variation or eligible omic data; discussion of genetic mechanisms or conventional drug/metabolite measurements alone was insufficient. Experimental opioid exposure of donor-derived human specimens was recorded separately from documented in vivo exposure. Retained reports, their PEOS basis, and qualifications were catalogued in Supplementary Table S5, together with the historical selection counts. Previously cited reports not returned by the strategies were listed separately and were not added to the search-yield count.

### 2.4. Data Extraction

Data were extracted using a customized relational workbook developed specifically for this review. The workbook comprised six interconnected modules linked through a unique study identifier: study-level characteristics, opioid exposure and toxicology, PGx, omics, integration and outcomes, and confounding and certainty-support information. This structure enabled preservation of the original study-level granularity while allowing reproducible linkage of clinical, toxicological, genetic, molecular, and methodological data.

Extracted variables included publication year, country, study design, setting, sample size, population characteristics, principal opioid, exposure context, co-exposures, biological matrices, toxicological methods, PGx targets, variants, inferred metabolic phenotypes, omics platforms, molecular features, pathways, outcome definitions, validation procedures, confounders, and principal study findings. Original quantitative results were retained on their reported scales, including odds ratios (ORs), risk ratios, hazard ratios, regression and β coefficients, mean differences, standardized effects, drug and metabolite concentrations, parent-to-metabolite and tissue-to-blood ratios, fold changes, log_2_ fold changes, correlation coefficients, R^2^, Q^2^, area under the receiver operating characteristic curve (AUC), sensitivity, specificity, accuracy, confidence intervals (CIs), raw *p*-values, multiplicity-adjusted *p*-values, and molecular-feature counts. Data extraction was performed independently by three reviewers. Disagreements were resolved through discussion and consensus.

### 2.5. Risk-of-Bias (RoB) Assessment

RoB was assessed using the appropriate JBI critical appraisal instrument for each study design, including the tools for case reports, case series, analytical cross-sectional, case–control, cohort, quasi-experimental studies, and randomized controlled trials (RCTs) [29,37,38,39,40]. Appraisal emphasized both conventional design-related limitations and issues particularly relevant to opioid-related toxicity and death, including exposure classification, therapeutic vs. illicit use, polysubstance exposure, sampling timing, biological matrix, analytical validation, PGx methodology, omics processing, incomplete clinical or forensic information, and cause-of-death attribution. RoB was assessed independently by two reviewers. Disagreements were resolved through discussion and consensus.

Primary appraisal was domain based. For descriptive presentation only, each item rated ‘Yes’ was assigned one point, whereas ‘No’ and ‘Unclear’ were assigned 0; items rated ‘Not applicable’ were excluded from the denominator. Percentages were summarized using author-defined low- (≥75%), moderate- (50–74%), or high- (<50%) risk categories. These thresholds are not JBI-endorsed global categories and did not replace item-level or domain-based judgments. They were not used to exclude studies, statistically weight results, alter effect estimates, or mechanically determine certainty. The complete item-level appraisal workbook is available through the OSF project.

### 2.6. Data Synthesis and Statistical Analysis

Because of the substantial methodological, clinical, biological, and analytical heterogeneity of the included studies, a formal meta-analysis was considered inappropriate. Evidence was therefore synthesized using a structured, effect-size-oriented narrative approach consistent with the JBI methodology for reviews of association and the synthesis without meta-analysis reporting guideline [29,41]. Studies were grouped into PGx, metabolomic/lipidomic, proteomic, transcriptomic/epigenetic, and integrated multi-omic domains. For each domain, we summarized study number, opioids and co-exposures, biological matrices, analytical platforms, principal findings, molecular pathways, direction of change, and associations with toxicity or death. Where available, allele frequencies, genotype and phenotype distributions, and reported genotype-outcome associations were tabulated.

The study—not the individual molecular estimate—constituted the primary analytical unit. Original quantitative estimands, including ORs, regression coefficients, concentrations, parent-to-metabolite ratios, fold changes, correlations, AUC, sensitivity, specificity, R^2^, Q^2^, CIs, and multiplicity-adjusted results, were retained on their original scales without cross-study standardization or pooling. Quantitative results were included only when their metric, direction, study attribution, and uncertainty could be reconstructed unambiguously. Descriptive summaries of commensurable estimands comprised minimum, median, interquartile range (IQR), and maximum; absolute, relative, and percentage differences were derived only when both group values were reported. Multiple estimates originating from the same study were treated as dependent observations and were not counted as independent replications.

Convergence and divergence across biomarker domains were explored qualitatively, together with heterogeneity by study design, biological matrix, geographical region, analytical platform, therapeutic vs. illicit exposure, classical opioids vs. fentanyl/nitazene analogues, and acute overdose vs. chronic toxicity. Nominally significant findings were distinguished from multiplicity-adjusted findings. Predictive or discriminative performance was interpreted in relation to its validation level (derivation, internal validation, or external validation).

### 2.7. Certainty of Evidence Assessment

Certainty was assessed using GRADE for outcome-focused bodies of association evidence, with a narrative Summary of Findings and detailed evidence profile (Supplementary Table S3) [42,43]. Following peer review, the original six-question framework was revised to distinguish four outcome-focused bodies from two structured narrative appraisals. The four bodies concern the following: (1) PGx associations with opioid toxicity or death; (2) discrimination of opioid exposure or adverse outcomes by molecular signatures; (3) molecular discrimination or explanatory attribution of opioid-related toxicity and death in forensic settings; and (4) reproducibility and transportability of molecular associations or signatures across independent settings. The fourth makes explicit the reproducibility and transportability component of the original synthesis-level question; it is not a global GRADE rating of the review. Incremental interpretative utility of molecular integration and the adequacy of confounder control are appraised separately without certainty grades: the former lacks a directly evaluated comparative benefit in the included evidence, whereas the latter describes study methods rather than an outcome. This revision to the appraisal framework does not represent a new eligibility criterion.

Because effect estimates were not pooled, GRADE judgments were applied to narratively synthesized bodies of evidence. The bodies of evidence were treated as non-randomized association evidence and initially rated at low certainty. Although three included investigations used randomized designs, genotype or biomarker status was not randomized; these studies therefore did not increase the starting certainty of the corresponding molecular bodies of evidence.

For each outcome-focused body, judgments considered risk of bias, inconsistency, indirectness, imprecision, and risk of missing evidence. Potential upgrading factors were large effects, dose–response relationships, and residual confounding likely to attenuate an association. The certainty profile records the rationale for each judgment without mechanically translating study-level directness, validation, or JBI summary categories into GRADE levels. The heterogeneous, non-pooled estimates did not permit funnel-plot or regression-based assessment of publication bias. Restrictions to published English-language evidence and exploratory reporting create a possibility of missing negative evidence, but do not establish its extent or direction. Publication bias was therefore not designated ‘strongly suspected’ or used for an additional downgrade solely on those grounds [44].

### 2.8. Forensic Interpretative Directness

Forensic interpretative directness is an author-defined descriptive classification of how closely a study’s population, exposure, biological material, endpoint, and interpretative aim correspond to postmortem toxicology or cause-of-death assessment. T.D. and A.M. assigned categories independently, resolving disagreements through discussion to a final agreed classification. No pre-consensus agreement statistic is available; the final consensus ratings cannot be used to reconstruct one. High directness requires an explicit link between human medicolegal, postmortem, or fatal-toxicological findings and molecular interpretation of toxicity or death. Moderate directness denotes severe toxicity or a clearly transferable PK/PD mechanism without validation for individual postmortem attribution. Low directness denotes therapeutic response, non-severe adverse effects, experimental PK, chronic exposure/OUD, or a non-specific signature requiring substantial extrapolation. No directness denotes no identifiable transfer to the forensic question. The complete operational rubric and study-level assignments are provided in Supplementary Methods S1, Section 2.3, and the extraction materials. Statistical significance, effect size, sample size, precision, replication, risk of bias, and GRADE certainty are not criteria for assigning directness.

The forensic orientation and recency, evidence-support, composite-validation, and coverage-gap scores are exploratory descriptive constructs based on internal normalization and author-defined coding and weights. The horizontal score *F* combines forensic-setting representation, multidimensional directness, and recency; evidence support *S* separately combines study count, molecular breadth, and composite validation. The coverage gap is max(0, *F* − *S*). Neither construct is calibrated to epidemiological burden, toxicity, cause-of-death adjudication, or clinical risk. Definitions and computational dependencies are provided in Supplementary Methods S1.

## 3. Results

### 3.1. Study Selection

**Figure 1:**
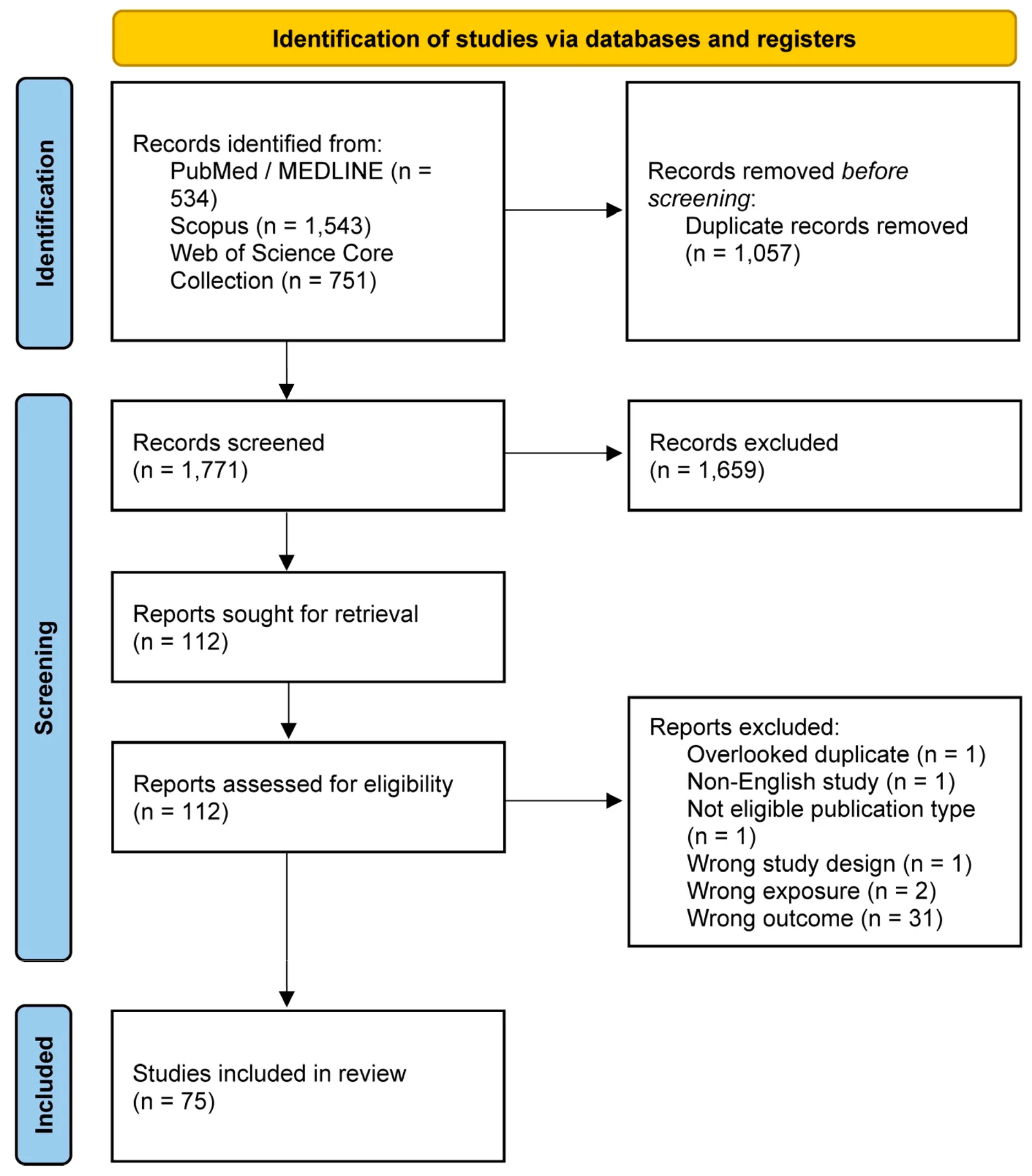
Study selection flow diagram according to PRISMA 2020 for the formal 2008–March 2026 review. The separate pre-2008 historical audit is reported in Section 3.1 and Supplementary Table S5 and is not incorporated into these counts.

The database search was completed in March 2026 and identified 2828 records: 534 from PubMed/MEDLINE, 1543 from Scopus, and 751 from the Web of Science Core Collection. After removal of 1057 duplicates, 1771 records underwent title-and-abstract screening, of which 1659 were excluded. All 112 reports sought for retrieval were obtained and assessed in full text. Thirty-seven of them were excluded because of an overlooked duplicate (*n* = 1), non-English publication (*n* = 1), ineligible publication type (*n* = 1), wrong study design (*n* = 1), exposure (*n* = 2), or outcome (*n* = 31), leaving 75 studies in the final synthesis [45,46,47,48,49,50,51,52,53,54,55,56,57,58,59,60,61,62,63,64,65,66,67,68,69,70,71,72,73,74,75,76,77,78,79,80,81,82,83,84,85,86,87,88,89,90,91,92,93,94,95,96,97,98,99,100,101,102,103,104,105,106,107,108,109,110,111,112,113,114,115,116,117,118,119] (Figure 1).

Nitazene records were retrieved but did not contribute to the eligible molecular corpus. A focused audit of the archived title/abstract screening file identified at least 10 records explicitly naming nitazenes or named nitazene derivatives in their titles; all were recorded as excluded before full-text assessment. The recorded reasons concerned outcomes, population, or study design. None of these records occurs among the 112 full texts. Supplementary Table S4 lists the records and original reasons; this transparent minimum count is not an exhaustive estimate of every record mentioning nitazenes anywhere in its metadata.

The supplementary historical search identified 414 records: 52 from MEDLINE/PubMed, 294 from Scopus, and 68 from Web of Science Core Collection. After removal of 112 duplicates, 302 records underwent title/abstract screening; 292 were excluded and 10 proceeded to full-text assessment. All ten full texts were retrieved and retained, with no full-text exclusions. Supplementary Table S5 identifies these reports as Bhasker (2000) [120], Jannetto (2002) [121], Lötsch (2002) [122], Levo (2003) [123], Gasche (2004) [34], Klepstad (2004) [124], Jin (2005) [125], Drakenberg (2006) [126], Koren (2006) [127], and Lee (2006) [128]. Nine concern in vivo exposure; Bhasker was retained under the audit’s human-biospecimen/experimental interpretation of PEOS and examined morphine glucuronidation in genotyped human liver microsomes. All ten directly investigated a PGx component. Study-level qualifications, including the Expression of Concern attached to Koren (2006) [127,129], are documented in Supplementary Table S5. Gasche was among the ten retrieved reports; Sindrup (1990) and Kirchheiner (2007) were not returned and are not included in that count [33,34,35]. Ten is therefore a documented, strategy-specific minimum under the stated PEOS interpretation, not an exhaustive historical estimate. These reports remain separate from the formal 75-study corpus and its selection flow.

### 3.2. Characteristics of the Included Studies

The 75 included studies were published between 2008 and 2026, with a median publication year of 2019 and an IQR of 2013–2022. The evidence base comprised 20 analytical cross-sectional studies (26.7%), 14 cohort studies (18.7%), 14 case–control studies (18.7%), 12 case reports (16.0%), 8 case series (10.7%), 4 quasi-experimental studies (5.3%), and 3 randomized studies (4.0%). Study populations ranged from individual clinical or fatal cases to large observational cohorts and biobank-based investigations. Table 1 provides a concise synthesis grouped by molecular evidence domain, highlighting study types, molecular targets, opioids, principal findings, and forensic relevance. Complete characteristics of all 75 studies, including study design, country, sample size, exposure context, biological matrix, analytical methods, outcomes, validation, and RoB, are reported in Supplementary Table S2 [45,46,47,48,49,50,51,52,53,54,55,56,57,58,59,60,61,62,63,64,65,66,67,68,69,70,71,72,73,74,75,76,77,78,79,80,81,82,83,84,85,86,87,88,89,90,91,92,93,94,95,96,97,98,99,100,101,102,103,104,105,106,107,108,109,110,111,112,113,114,115,116,117,118,119].

Therapeutic exposure represented the most frequent context, accounting for 29 studies (38.7%), followed by fatal intoxication in 15 studies (20.0%), OUD or chronic exposure in 9 studies (12.0%), postmortem opioid detection in 8 studies (10.7%), experimental administration in 6 studies (8.0%), illicit exposure in 4 studies (5.3%), non-fatal overdose in 2 studies (2.7%), misuse in 1 study (1.3%), and an unclear exposure context in 1 study (1.3%) [45,46,47,48,49,50,51,52,53,54,55,56,57,58,59,60,61,62,63,64,65,66,67,68,69,70,71,72,73,74,75,76,77,78,79,80,81,82,83,84,85,86,87,88,89,90,91,92,93,94,95,96,97,98,99,100,101,102,103,104,105,106,107,108,109,110,111,112,113,114,115,116,117,118,119].

PGx studies constituted the largest molecular domain in 51 studies (68.0%). A further 11 studies primarily investigated transcriptomic or epigenetic markers (14.7%), 3 investigated metabolomic signatures (4.0%), 1 investigated proteomics (1.3%), 2 applied integrated multi-omic approaches (2.7%), and 7 addressed other or incompletely classifiable forms of molecular evidence (9.3%). Biological matrices included whole blood, plasma, serum, urine, hair, placenta, brain tissue, medulla, liver, postmortem fluids, and multi-matrix combinations. Analytical methods ranged from candidate-gene genotyping and copy-number assessment to sequencing, liquid chromatography–tandem mass spectrometry (LC-MS/MS) toxicology, nuclear magnetic resonance (NMR) spectroscopy, liquid chromatography–high-resolution mass spectrometry (LC-HRMS) metabolomics, methylation arrays, RNA sequencing, chromatin immunoprecipitation sequencing (ChIP-seq), proteomic mass spectrometry, and integrated network analyses [45,46,47,48,49,50,51,52,53,54,55,56,57,58,59,60,61,62,63,64,65,66,67,68,69,70,71,72,73,74,75,76,77,78,79,80,81,82,83,84,85,86,87,88,89,90,91,92,93,94,95,96,97,98,99,100,101,102,103,104,105,106,107,108,109,110,111,112,113,114,115,116,117,118,119].

RoB assessment classified 36 studies as low risk, 36 as moderate risk, and 3 as high risk, corresponding to 48.0%, 48.0%, and 4.0% of the evidence base, respectively. Moderate-risk judgments most frequently reflected limited sample size, incomplete control of co-medications or polysubstance exposure, retrospective exposure reconstruction, incompletely characterized ancestry, indirect outcome definitions, or limited validation. No study was excluded solely because of its RoB classification.

### 3.3. Multidimensional Distribution of the Evidence

**Figure 2:**
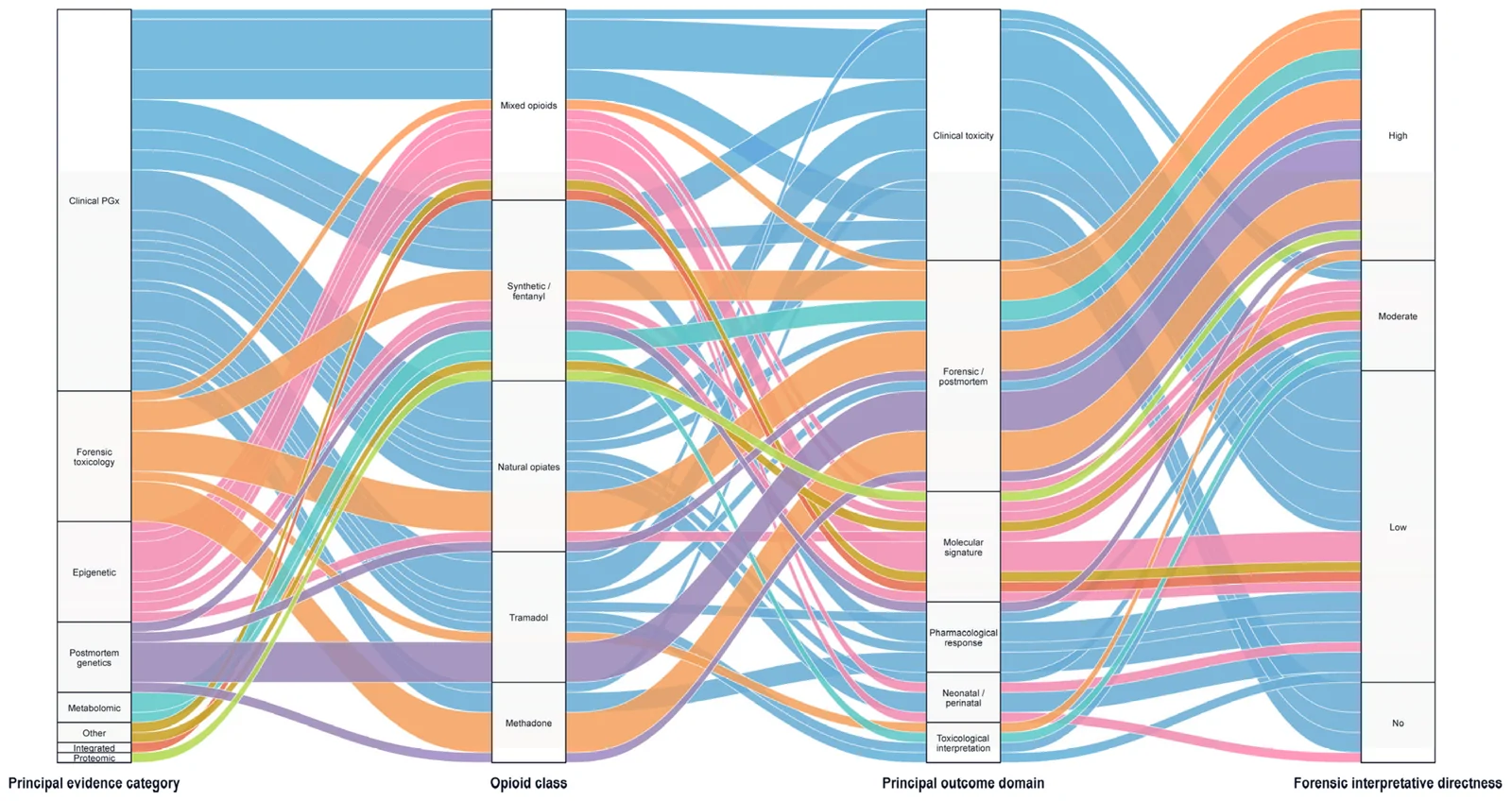
Study-level distribution of the included evidence. Each of the 75 studies contributes one pathway through principal evidence category, opioid class, outcome domain, and forensic interpretative directness. Flow colors correspond to the principal evidence category: light blue, Clinical PGx; orange, Forensic toxicology; pink, Epigenetic; purple, Postmortem genetics; teal, Metabolomic; ochre, Other; green, Integrated; and coral red, Proteomic. Colors are retained throughout each pathway for visual tracking and do not encode study weight, quality, certainty, or evidential strength. Band widths and labels report study counts; categories within each axis are mutually exclusive, and small nodes include padding for readability. A case report and a large cohort each contribute one pathway: the figure is not weighted by participants, precision, design, or evidential strength. Twenty studies (26.7%) are case reports or case series. These display categories differ from the molecular-domain coding in Table 1. Directness is an author-defined descriptive classification, distinct from risk of bias, certainty, and validation. The figure describes the eligible 2008–March 2026 literature; definitions and assignments are provided in Supplementary Methods S1.

The multidimensional distribution of the evidence is presented in Figure 2. Each pathway represents a unique study, and band widths encode study counts. Clinical PGx constituted the dominant principal evidence category, while mixed opioids, synthetic/fentanyl, and natural opiates were the most represented exposure classes. Clinical-toxicity and forensic/postmortem outcomes formed the broadest outcome strata. Forensic interpretative directness was high in 25 studies (33.3%), moderate in 11 (14.7%), low in 31 (41.3%), and absent in 8 (10.7%); thus, 39 of 75 studies (52.0%) had low or no directness to forensic interpretation.

**Figure 3:**
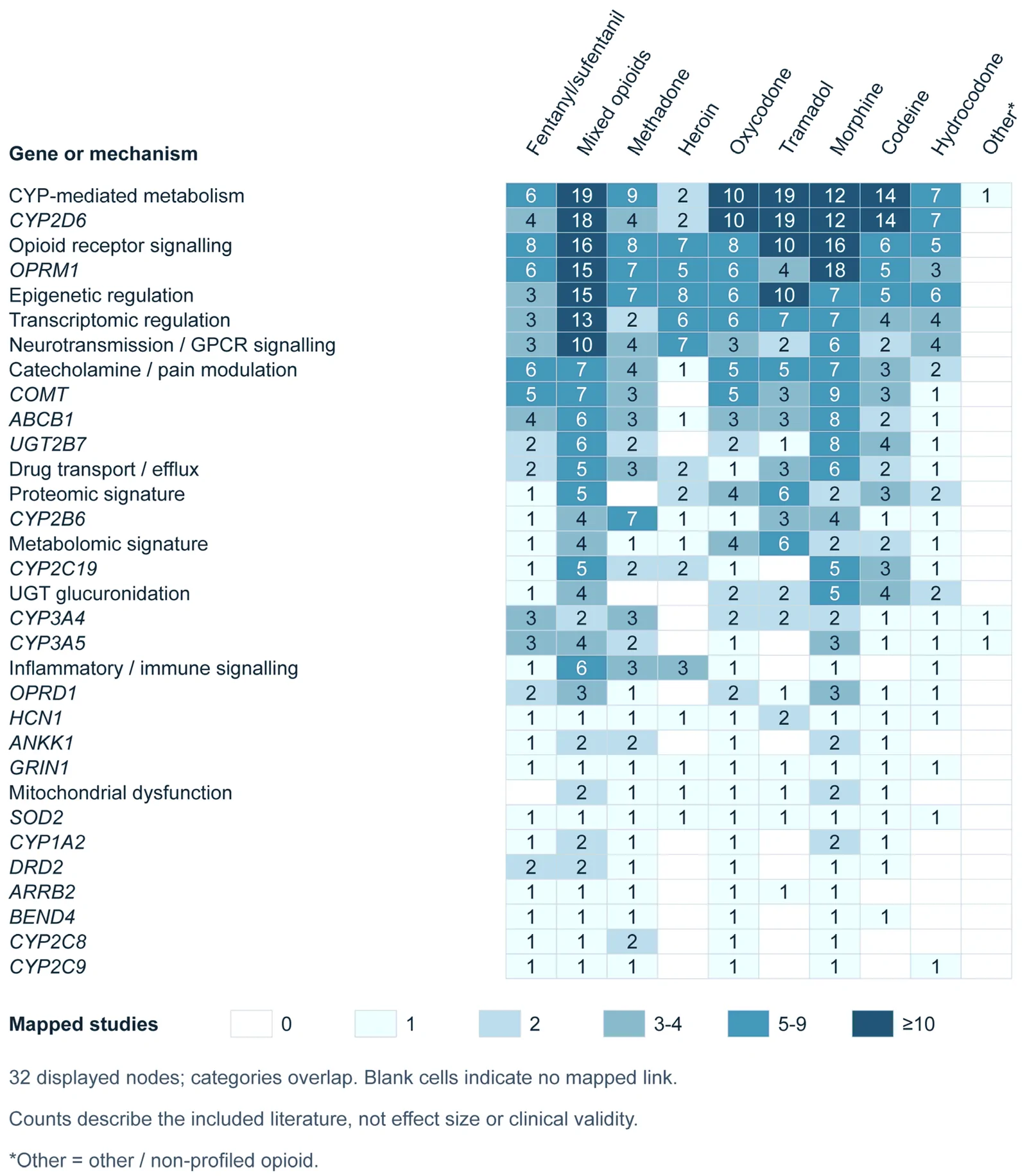
Molecular-opioid co-occurrence in the included studies. Cells report the number of unique studies linking each opioid category to each of the 32 most frequently mapped molecular nodes. A study may contribute to multiple opioid categories and nodes; counts therefore overlap and must not be summed as independent studies. Links indicate co-occurrence in study-level ex-traction records, not necessarily an opioid-specific association or a causal relationship. The matrix distinguishes canonical genes from broader mechanism and omics categories. Blank cells indicate no mapped link among included studies. Complete extraction and ranking rules are provided in Supplementary Methods S1.

The study-level molecular co-occurrence matrix (Figure 3) shows that connectivity was concentrated around CYP-mediated metabolism, particularly CYP2D6, with additional recurrent links involving opioid-receptor signaling, OPRM1, COMT, ABCB1, UGT2B7, and epigenetic and transcriptomic regulation. Mixed-opioid studies formed the broadest molecular representation, while fentanyl/sufentanil, methadone, tramadol, codeine, and morphine showed the richest connectivity among the principal opioid categories [45,46,47,48,49,50,51,52,53,54,55,56,57,58,59,60,61,62,63,64,65,66,67,68,69,70,71,72,73,74,75,76,77,78,79,80,81,82,83,84,85,86,87,88,89,90,91,92,93,94,95,96,97,98,99,100,101,102,103,104,105,106,107,108,109,110,111,112,113,114,115,116,117,118,119]. Links indicate co-occurrence within the same study record and do not demonstrate opioid-specific effects or causal relationships, particularly in mixed-opioid studies.

### 3.4. Quantitative Architecture of the Narrative Synthesis

The extraction pass over the workbook fields identified at least one numerical occurrence for 66 of 75 studies (88.0%). The remaining nine did not yield a numerical occurrence in that extraction pass; this does not imply that their original reports contained no quantitative findings [45,51,66,78,97,100,108,118,119]. Automated extraction initially identified 606 numerical occurrences. After removal of repeated reporting within and across workbook fields, 332 unique estimands remained. Semantic adjudication subsequently excluded sample sizes, publication years, variant identifiers, analytical thresholds, isolated *p*-values, and values whose metric or meaning could not be reconstructed, leaving 124 substantive estimands.

A subset of 114 estimands from 43 studies met the requirements for structured cross-study presentation. Their distribution across opioids and the highest validation tier assigned to each study are presented in Supplementary Figure S1 and Table 2. Multiple estimates from a study remain dependent; the 114 estimands do not represent 114 independent investigations.

Within the 43-study quantitative subset, 29 studies remained at discovery/descriptive analysis, three had internal/technical validation, five used cross-validation, and six had external/independent validation (Table 2). These tiers describe validation of the reported analysis and do not establish diagnostic validity for opioid-related death.

### 3.5. CYP2D6-Mediated Bioactivation and Opioid Response

The CYP2D6 axis generated the greatest number of quantitatively interpretable association effects. Across four studies contributing 10 extractable ORs within the CYP2D6-bioactivation cluster, the unweighted median OR was 6.21, with an IQR of 2.68–8.40 and a range of 2.00–16.50. A total of 5 estimates had extractable CIs. Their median relative CI width was approximately 1.07 times the point estimate, whereas the widest interval was substantially larger than the estimate itself. These values describe the observed distribution and do not constitute a pooled CYP2D6 effect because the underlying estimates concerned different opioids, outcomes, genetic models, and populations [47,112,116].

In a real-world cohort of 219 analyzable opioid recipients, CYP2D6 poor or ultrarapid metabolizers had 2.68-fold higher adjusted odds of poor pain control or an opioid-related adverse reaction than intermediate-to-extensive metabolizers (95% CI, 1.39–5.17; *p* = 0.003) [47]. The association persisted after exclusion of CYP2D6-inhibitor users, although it attenuated when the analysis was restricted to oxycodone and hydrocodone.

Maternal–neonatal codeine studies produced larger but less precise estimates. Symptomatic mothers used a 59% higher weight-adjusted codeine dose than asymptomatic mothers (*p* = 0.004), while the combined CYP2D6 ultrarapid-metabolizer/UGT2B7*2/*2 genotype was associated with infant CNS depression with an OR of 8.40 (95% CI, 4.70–47.00; *p* ≤ 0.001) [112]. In the multilocus analysis, maternal ABCB1 2677TT was associated with infant CNS depression with an OR of 6.21 (95% CI, 2.27–17.02), maternal CYP2D6 ultrarapid-metabolizer status with an OR of 16.50 (95% CI, 1.09–250.18), and the multilocus score with an OR of 2.68 per unit increase (95% CI, 1.61–4.48). The combined genetic and clinical model achieved an AUC of 0.87 (95% CI, 0.75–0.98), with 80% sensitivity and 87% specificity [116]. The extremely wide interval surrounding the ultrarapid-metabolizer estimate indicated that its positive direction was more stable than its exact magnitude.

### 3.6. CYP2D6-Mediated Codeine Bioactivation: Genotype, Phenoconversion, and Realized Phenotype

Among 34 codeine-related deaths, mean morphine-to-codeine ratios were 0.058 in extensive metabolizers, 0.043 in intermediate metabolizers, and 0.025 in poor metabolizers [46]. Relative to extensive metabolizers, the mean ratio was 25.9% lower in intermediate metabolizers and 56.9% lower in poor metabolizers. However, the phenotype-specific ranges overlapped substantially: 0–0.39 in extensive metabolizers, 0–0.22 in intermediate metabolizers, and 0.005–0.044 in poor metabolizers. Across all cases, codeine concentrations ranged from 0.30 to 15.0 mg/L; morphine ranged from not detectable to 3.1 mg/L, morphine-6-glucuronide from not detectable to 0.55 mg/L, and morphine-3-glucuronide from not detectable to 1.5 mg/L. The overall mean morphine-to-codeine ratio was 0.054, with a range of 0–0.39 and a standard deviation (SD) of 0.087. Codeine and morphine concentrations were only weakly related (R^2^ = 0.21). A total of 13 cases had codeine concentrations above 1.6 mg/L, but 8 of these had morphine below the toxic range. A total of two low ratios in extensive-metabolizer genotypes occurred during exposure to CYP2D6 inhibitors, supporting phenoconversion [46].

In controlled codeine administration, genotype, phenotyping, and combined genotype-plus-phenotype systems, each identified 87.5% of subjects with very low morphine formation. For extremely high morphine formation, genotype alone correctly classified 50.0%, phenotyping 62.5%, and the combined approach 87.5% [102]. Integration therefore improved classification by 37.5 percentage points compared with genotype alone.

In 55 codeine-related deaths, increasing CYP2D6-inhibitor potency was associated with progressively lower morphine-to-codeine ratios (*p* = 0.0011). Deaths involving additional CNS depressants occurred at lower codeine concentrations than codeine-only deaths (*p* = 0.0002). ABCB1 1236T carriers had a median morphine concentration of 0 ng/mL compared with 17 ng/mL among 1236CC individuals (*p* = 0.004), whereas CYP2D6 genotype alone was not independently predictive in multivariable analysis [113].

Case reports illustrated mechanistically informative extremes. In a driver with confirmed codeine use, intermediate CYP2D6 metabolism, ultrarapid UGT2B7 glucuronidation, and CYP2D6 inhibition explained free morphine of 0 μg/L despite free codeine of 348 μg/L and a total morphine-to-codeine ratio of 2.4% [95]. Severe intoxication in pediatric twins was more consistent with dosing error, continued absorption, and redistribution than with ultrarapid metabolism [111]. A CYP2D6*4/*5 poor-metabolizer genotype supported reinterpretation of suspected codeine overdose as inadequate morphine formation and ineffective analgesia [118]. Familial CYP2D6 duplication demonstrated transmission of an ultrarapid-metabolizer genotype in a breastfeeding context [97]. Overall, the codeine evidence supported a coherent metabolic mechanism but also showed substantial dependence on dose, age, glucuronidation, co-medications, breastfeeding exposure, and clinical circumstances [46,95,97,100,102,111,112,113,116,118].

### 3.7. CYP2D6 and Tramadol: Metabolic Ratios Are More Informative than Absolute Concentrations

In a phase II therapeutic study, mean neuropathic-pain improvement was −1.50 in intermediate metabolizers and −0.67 in extensive metabolizers, yielding an unadjusted between-phenotype difference of −0.83 points; toxicity-related discontinuation occurred in 33% and 16%, respectively, corresponding to an absolute difference of 17 percentage points and an approximate risk ratio of 2.06. The genotype subgroup comprised only 25 patients and included no poor or ultrarapid metabolizers [45].

A near-fatal tramadol overdose in a CYP2D6*1/*1×2 ultrarapid metabolizer produced an admission concentration of 3.22 mg/L, a tramadol-to-O-desmethyltramadol ratio of 2.54, and a tramadol-to-N-desmethyltramadol ratio of 11.4 [48]. Concomitant CYP3A4 and CYP2B6 inhibition was interpreted as redirecting metabolism toward CYP2D6-mediated O-desmethyltramadol formation. Another ultrarapid metabolizer with renal impairment showed (+)-O-desmethyltramadol concentrations approximately two-fold higher at 30 min and three-fold higher at 3 h than other ultrarapid metabolizers, followed by delayed respiratory depression completely reversed by naloxone [119].

Among 74 tramadol-dependent patients, OPRM1 A118G and ABCB1 C3435T were not associated with seizure status (*p* = 0.50 and *p* = 0.17). By contrast, prior head trauma occurred in 24.3% of seizure cases and 0% of controls (*p* = 0.016), while opioid-containing antitussive exposure occurred in 20% and 0%, respectively (*p* = 0.036) [49].

Postmortem metabolic-ratio studies were more consistent than analyses of absolute tramadol concentrations. One study found no robust association between CYP2D6 genotype and absolute concentrations [55]. In contrast, M2/M1 differentiated poor from extensive or intermediate metabolizers at *p* < 0.01 [56]. CYP2D6 rs35742686 was associated with M2/M1 and CYP3A4 rs35599367 with MR2, both at *p* < 0.01; CYP2D6 rs1058172 and CYP2B6 rs4803419 also differed between intoxication and non-intoxication groups at *p* = 0.02 and *p* = 0.04 [64]. A total of 12 of 73 variants in UGT1A8, ABCC2, and SLC22A1 were associated with MR1, MR2, or M2/M1 at *p* = 0.001–0.038, whereas no UGT2B7 variant was significant and no locus was overrepresented in fatal cases [60].

CYP2D6 sequencing in 97 postmortem tramadol cases identified 6 poor metabolizers. An N-desmethyltramadol/O-desmethyltramadol ratio above 7 separated all 6 poor metabolizers from non-poor metabolizers, and 5 of the 6 had tramadol concentrations above 800 ng/mL. Cases involving enzyme inhibitors occupied an intermediate ratio range of approximately 2.5–7 [101]. Although separation was complete within this dataset, external sensitivity, and specificity were not reported.

Hair analysis demonstrated a monotonic phenotype gradient. Mean O-desmethyltramadol concentrations were 92.0 pg/mg in wild-type homozygotes, 31.0 pg/mg in *10/*10 subjects, and 14.0 pg/mg in *5/*5 subjects. N-desmethyltramadol increased from 31.7 to 88.3 and 142.5 pg/mg, while O-desmethyltramadol/tramadol ratios were 0.13, 0.04, and 0.02 [87]. Relative to wild-type subjects, O-desmethyltramadol was 66.3% lower in *10/*10 subjects and 84.8% lower in *5/*5 subjects. These large gradients did not reach conventional significance because of the small phenotype strata.

A total of 14 variants met a Bonferroni threshold of 2.42 × 10^−6^ in a pathway-driven analysis. UGT2B7 variants accounted for an estimated 82.1% of tramadol-to-M1 ratio heritability (*p* = 1.22 × 10^−6^), 33 variants explained 32.9% of ratio variability, and a 16-variant, 5-gene model achieved classification accuracy up to 93.8%, and outperforming CYP2D6-only prediction (paired *p* = 0.019) [90]. A related model reported 96.3% apparent accuracy for an unphased random forest and 52.3% for phased regularized multinomial regression. A total of seven loci were selected from 1875 candidates, corresponding to retention of approximately 0.37% of candidate loci, although genotype–phenotype discordance remained highly significant (*p* = 1.57 × 10^−31^) [91]. A separate genome-wide association study (GWAS) after false-discovery-rate (FDR) adjustment identified 5 FDR-adjusted genome-wide associations with adjusted *p*-values between 1.70 × 10^−11^ and 5.48 × 10^−9^, but none replicated in an independent cohort of 99 cases [76].

Long-term tramadol treatment produced large biochemical contrasts. Alanine aminotransferase was 189.82 ± 15.45 U/L in CYP2D6-duplication carriers and 37.51 ± 4.28 U/L in wild-type controls, an absolute difference of 152.31 U/L and a 5.06-fold ratio. Aspartate aminotransferase was 268.03 ± 18.87 vs. 31.14 ± 4.71 U/L, corresponding to an absolute difference of 236.89 U/L and an 8.61-fold ratio; both comparisons had *p* < 0.0001. Grade ≥ 2 hepatotoxicity occurred in 21 of 49 duplication or *1 carriers and 0 of 11 *4/*10 carriers, an absolute risk difference of 42.9 percentage points (*p* = 0.0049). M1 correlated with alanine aminotransferase at *r* = 0.97 and with α-glutathione-S-transferase at *r* = 0.93. M1 and malondialdehyde remained independently associated with α-glutathione-S-transferase, with β = 0.03245 (*p* = 0.0163) and β = 1.77084 (*p* = 0.0074), respectively [84].

The principal CYP2D6- and tramadol-related estimates are summarized in Table 3.

### 3.8. Methadone: CYP2B6 Disposition and ABCB1-Related Tissue Distribution

Controlled methadone administration showed S-methadone apparent oral clearance approximately 35% lower in CYP2B6*1/*6 carriers and 45% lower in *6/*6 carriers than in *1/*1 individuals. Corresponding reductions for R-methadone were approximately 25% and 35%, while *4 carriers had three- to four-fold higher apparent oral clearance. Clearance correlated with N-demethylation at *r* = 0.57 for R-methadone and *r* = 0.82 for S-methadone, both *p* < 0.001 [99].

In methadone fatalities, CYP2B6 rs3745274 and rs8192719 minor-allele frequencies were 31.9% and 33.6%, compared with 22.4% and 23.0% in controls, with *p* = 0.0012 and *p* = 0.00052. Homozygous rs3211371 carriers had mean methadone concentrations of 1.67 ± 0.85 mg/L compared with 0.59 ± 0.05 mg/L in ancestral-genotype carriers, an absolute difference of 1.08 mg/L and a 2.83-fold ratio (*p* = 0.002) [80].

In another fatality series, mean methadone concentration was 0.93 mg/L in *1/*6 and 0.56 mg/L in *1/*1 subjects, an absolute difference of 0.37 mg/L and a relative increase of 66.1% (*p* = 0.041). OPRM1 118GA carriers had 2.4-fold higher benzodiazepine concentrations than 118AA carriers (*p* = 0.004) [106]. A subsequent study found methadone concentrations of 0.96 and 0.58 mg/L by CYP2B6 group, a difference of 0.38 mg/L, and a relative increase of 65.5% (*p* = 0.039) [117]. In living patients, CYP2B6 loss-of-function alleles were associated with a 0.294-unit reduction in log S-EDDP/methadone, corresponding to a 1.342-fold lower ratio (*p* = 0.012) [81].

ABCB1 rs1045642 was associated with a large tissue-distribution effect. TT homozygotes had higher medulla-to-blood ratios than CT/TC carriers, with Cohen’s *d* = 1.54 (95% CI, 1.14–2.05; *p* = 0.002), and higher ratios than CC homozygotes, with *d* = 1.60 (95% CI, 1.13–2.29; *p* = 0.004). Overall analysis of variance (ANOVA) *p* was 0.001. The mean medulla-to-blood ratio was 2.85, SD 1.83, and range 0.71–11.67, despite a mean femoral-blood methadone concentration of 0.74 mg/L [79].

CYP3A4 evidence was less consistent. Selected variants were enriched in methadone-fatality subgroups, including *p* = 0.02 for rs2242480 and *p* = 1.2 × 10^−22^ for rs2740574, but methadone concentrations did not differ significantly by genotype, and deviations in control allele distributions limited interpretation [114]. CYP3A4*1B was associated with improved withdrawal control in a PGx-guided cohort, with OR 6.646 (95% CI, 1.208–36.553; *p* = 0.0294), while time to first withdrawal differed by phenotype (log-rank *p* = 0.015) [75].

### 3.9. Fentanyl/Sufentanil and the Contemporary Synthetic-Opioid Evidence Gap

Among 821 opioid-exposed emergency-department patients, CYP3A5 rs776746 CC was associated with non-fatal overdose with an OR of 6.96 (95% CI, 2.45–29.23), and TC with an OR of 4.27 (95% CI, 1.38–18.75). DRD2 rs4436578 AA was associated with an OR of 5.58 (95% CI, 1.93–23.67), whereas NK1R rs6715729 CC was inversely associated with overdose (OR 0.28; 95% CI, 0.14–0.54). The principal signals survived Bonferroni correction, with adjusted *p*-values between 3.78 × 10^−6^ and approximately 1.5 × 10^−4^ [67].

Among hospitalized patients requiring naloxone, CYP3A5 genotype and phenotype distributions differed between cases and controls at *p* = 0.004 and *p* = 0.038, while CYP2D6, CYP3A4, and UGT2B7 did not. Mean opioid exposure during the preceding 24 h was 231.3 ± 228.3 vs. 94.1 ± 96.1 morphine-milligram equivalents, a difference of 137.2 morphine milligram equivalents (MME) [71].

In a PK pilot study of fentanyl, plasma concentration at 15 min was 3.2 ± 0.2 ng/mL in 3 variant carriers and 1.5 ± 0.5 ng/mL in wild-type patients, corresponding to an absolute difference of 1.7 ng/mL and a 2.13-fold ratio (*p* < 0.001). Reported clearance was 4.67–9.13 mL/min in variant carriers vs. a median of 1115.6 mL/min in wild-type patients [85].

In a multicenter randomized study, CYP3A4 rs2242480 GA/AA carriers received 87.6% of the standard sufentanil concentration. Analgesic efficacy remained equivalent, while vomiting decreased from 6.85% to 0.54%, an absolute reduction of 6.31 percentage points, and urinary retention decreased from 12.33% to 2.17%, an absolute reduction of 10.16 percentage points [63]. Conversely, ABCB1 C1236T and C3435T did not significantly alter fentanyl-associated respiratory rate or oxygen saturation after multiplicity correction [109].

### 3.10. Pharmacodynamic and Polygenic Variability Beyond CYP-Mediated Metabolism

In advanced cancer, the COMT rs4680 A allele was associated with lower morphine-equivalent dose, while the adjusted odds of a sickness-response phenotype were 7.10 (95% CI, 1.51–33.41; *p* = 0.01). The combined COMT AG/OPRM1 AA profile was associated with greater pain (adjusted OR 1.55; *p* = 0.04), greater nausea (adjusted OR 5.47; *p* = 0.02), and less drowsiness (adjusted OR 0.25; *p* = 0.05) [50].

In women undergoing total knee replacement, OPRM1 118GG carriers consumed 22.9 mg vs. 15.0 mg morphine at 24 h, an absolute difference of 7.9 mg and a relative increase of 52.7%. At 48 h, consumption was 40.8 vs. 24.4 mg, an absolute difference of 16.4 mg and a relative increase of 67.2%; both comparisons had *p* = 0.04 [83]. Combined OPRM1 and COMT carriers in another postoperative cohort required 51.9 ± 12.1 mg vs. 71.6 ± 44.7 mg morphine, an absolute difference of −19.7 mg [103].

UGT2B7 802CC patients required 12 ± 9 mg morphine compared with 37 ± 22 mg in TT patients, an absolute reduction of 25 mg and a relative reduction of 67.6% (*p* = 0.005). A UGT2B7/OPRM1/ABCB1 model explained approximately 30% of morphine-dose variability [115].

In prescription-opioid-use disorder, OPRM1 AA carriers had a median of seven adverse events vs. five in AG/GG carriers (*p* = 0.046), and nausea occurred in 33% vs. 0% (*p* = 0.034). COMT homozygotes had a median of eight adverse events (*p* = 0.026), while OPRD1 and ARRB2 variants were associated with selected adverse effects [92].

In obstructive sleep apnea, morphine did not significantly increase the average primary hypoxemia outcome, but the OPRM1 genotype-by-treatment interaction was significant (*p* = 0.014). Hypercapnic ventilatory response decreased by −0.90 L/min/mmHg in AG carriers (95% CI, −1.46 to −0.33; *p* = 0.0041), compared with −0.15 in AA carriers (*p* = 0.24) [94].

In a large investigation of persistent opioid use, 6 of 10 OPRM1 signals were nominally significant and 2 survived Bonferroni correction, whereas OPRD1 and DRD2/ANKK1 findings did not replicate [52]. The largest OR, 18.17, involved a KCNN1 variant with a minor-allele frequency of approximately 0.0001.

A GWAS of opioid-associated nausea identified 65 variants at *p* < 1 × 10^−5^, but none reached the conventional genome-wide threshold of *p* < 5 × 10^−8^. The leading variant had β = −4.34 at *p* = 7.4 × 10^−8^. Female sex had β = 5.5 (*p* = 3.9 × 10^−5^), age β = −0.12 (*p* = 0.009), and buprenorphine vs. morphine β = −6.2 (*p* = 0.026) [73].

The principal quantitative effects beyond CYP2D6 are detailed in Table 4. A complementary interpretative overview of the pharmacogenetic evidence is provided in Table 5, highlighting the greater coherence of CYP2D6-related codeine and tramadol metabolism, CYP2B6-related methadone disposition, and ABCB1-related tissue distribution, alongside the heterogeneity of pharmacodynamic associations and the sparse evidence for contemporary synthetic opioids.

### 3.11. Metabolomic and Preliminary Proteomic Findings

The postmortem oxycodone metabolomic study identified 25 reduced acylcarnitines, with fold changes of approximately 0.46–0.79 and Bonferroni-adjusted *p* < 0.001 [70]. These values represented reductions of approximately 21–54%. The orthogonal partial least-squares discriminant analysis (OPLS-DA) model had R^2^ = 0.41 and Q^2^ = 0.21, producing an R^2^-Q^2^ difference of 0.20. Training sensitivity and specificity were both 80%. In external validation, sensitivity remained 80%, while specificity decreased to 74%.

Untargeted urinary fentanyl metabolomics analyzed 186 variables. A reversed-phase soft independent modeling by class analogy (SIMCA) model using four principal components achieved 96% sensitivity and 74% specificity under 10-fold cross-validation, while the hydrophilic interaction liquid chromatography (HILIC) model achieved 100% sensitivity and 76% specificity [78]. These models classified fentanyl exposure rather than fatal intoxication.

In a fatal infant methadone intoxication, blood methadone concentration was 570 ng/mL, and the first three principal components explained 63.3% of urinary spectral variance. The case clustered with non-surviving perinatal-asphyxia cases rather than with healthy controls or surviving asphyxia cases [59].

Across the two metabolomic classification studies, sensitivity ranged from 80% to 100% and specificity from 74% to 80% [70,78]. These estimates were not directly commensurable because they concerned different opioids, matrices, outcomes, and validation designs.

Proteomic evidence is limited to one exploratory postmortem study of 24 heroin users and 24 controls. It identified 288 differentially expressed proteins (≥2-fold change; FDR ≤ 0.01), distributed across hippocampus (87), putamen (121), and caudate (80), without a diagnostic-performance estimate or external validation [69]. This observation is retained at study level in Table 6 and does not establish a replicated proteomic signature or a domain-level forensic conclusion.

### 3.12. Transcriptomic, Epigenetic, and Integrated Analyses

A postmortem H3K27ac study of 51 overdose decedents and 51 matched controls identified 388 hypoacetylated regions and no hyperacetylated regions. A total of five convergent genes had FDR-adjusted *p*-values between 1 × 10^−11^ and 1 × 10^−6^. A seven-peak gradient-boosting model achieved a five-fold cross-validated AUC of 0.972 ± 0.037, and 108 resampled models had median AUCs above 0.90 [53].

Orbitofrontal-cortex profiling identified 397 differentially methylated CpGs mapping to 357 genes and 1740 differentially hydroxymethylated CpGs mapping to 1453 genes [54]. The hydroxymethylation feature count was 4.38-fold the methylation count. The 5hmC gene set was enriched for opioid-use-disorder GWAS signals with OR 1.8757 (*p* = 0.0028), whereas the 5mC set had OR 1.4371 (*p* = 0.2758). Agreement with an independent neuronal reference reached R^2^ = 0.66.

In acute opioid intoxication, no CpG survived FDR correction. The leading CpG had raw *p* = 2.07 × 10^−6^ but adjusted *p* = 0.405. Horvath age acceleration was null (*p* = 0.84), while PhenoAge acceleration was β = 2.24 years, standard error (SE) = 1.11, and *p* = 0.045 [82].

OPRM1 genotype–epigenotype interaction was associated with approximately 50% lower mRNA and 60% lower signaling in heroin-exposed 118G carriers, with *p* = 0.013 and *p* = 0.044. Methylation at +117 was 5.1 ± 1.7% vs. 2.1 ± 1.7%, an absolute difference of 3.0 percentage points (*p* = 0.0036), while at +145 it was 9.6 ± 4.6% vs. 3.3 ± 2.6%, a difference of 6.3 points (*p* = 0.029). Genotype-by-heroin interactions were significant for receptor density, F(1,61) = 10.9, *p* = 0.002, and mRNA, F(1,41) = 5.96, *p* = 0.019 [107].

Morphine-treated postmortem-derived neurons showed 166 upregulated and 146 downregulated genes using an exploratory threshold of FDR < 0.20 and an absolute fold change > 1.5, yielding 312 differentially expressed genes. For integration with an independent postmortem OUD dataset, the authors broadened the criterion to nominal *p* < 0.05 while retaining an absolute fold change > 1.5; this identified 340 upregulated and 236 downregulated genes. Of these, 20 overlapped with the postmortem dataset and 11 were directionally concordant. Epigenetic-clock and transcriptional-maturity measures correlated at r = 0.69 (95% CI, 0.34–0.87; *p* < 0.001) [57].

Brain and blood miRNA-mRNA profiling identified 402 differentially expressed brain genes, 89 brain miRNAs, and 104 blood miRNAs. A total of 79 target genes overlapped, with hypergeometric *p* = 1.92 × 10^−20^. Only miR-340-5p showed a significant direct brain-blood correlation (*r* = −0.46; *p* = 0.02) [58].

Integrated methylation-expression analysis identified 272 genes overlapping hypomethylated and upregulated modules, with *p* = 8.42 × 10^−21^. Principal module coefficients were β = −0.18 and β = 0.16, both FDR-adjusted *p* = 0.05 [86]. Plasma exosomal profiling showed directional upregulation of 98% of an 84-miRNA panel across heroin and/or HIV groups. A total of four miRNAs were significantly increased in the combined heroin-plus-HIV group, with *p*-values between <0.001 and 0.010, whereas heroin-only and HIV-only effects were not significant [77].

H3K27 acetylation at GRIA1 correlated with years of heroin use at *r* = 0.70 (*p* = 0.0381) and inversely with urinary morphine at *r* = −0.47 (*p* = 0.0241). In the translational component, JQ1 reduced heroin self-administration by 53% on day 1, 36% on day 2, and cue-induced seeking by 42% [88].

ELK1 protein was 133.26 ± 8.21% of the control, BAG1 mRNA 80.46 ± 3.61%, and USE1 92.09 ± 2.76%. ELK1 protein correlated with urinary 6-monoacetylmorphine at R^2^ = 0.70, while genotype-specific associations reached R^2^ = 0.95 in a small subgroup [98].

Placental methylation studies identified 69 SLC transporters, 11 ABC transporters, and 7 protein-kinase-C genes at FDR < 0.05. Individual AUCs were 0.89 for SLC19A2 (95% CI, 0.80–0.97), 0.88 for SLC9A5, 0.85 for SLC25A35, and 0.84 for SLC16A3 [72]. A subsequent study identified 111 pain-related CpGs, with methylation differences from −10.62% to +10.40% and AUCs from 0.57 to 0.92. IL6ST reached an AUC of 0.92, NF1 0.91, CAPN1 0.89, and OPRM1 0.62. The leading CFTR result had raw *p* = 9.67 × 10^−39^ and FDR = 8.36 × 10^−33^ [68].

The integrated multi-omic network study identified 211 connected genes, 50 high-confidence genes, 48 druggable targets, and 414 candidate compounds. The five-fold cross-validated AUC was 0.94 [65]. The principal quantitative omics findings are detailed in Table 6. A complementary layer-specific overview is provided in Table 7, summarizing the potential interpretative contribution, principal limitations, and current maturity of the multi-omic evidence.

### 3.13. Certainty of Evidence

Certainty was very low for the four outcome-focused bodies assessed with GRADE (Supplementary Table S3). Recurrent concerns were risk of bias, inconsistency, indirectness, and imprecision, particularly the limited independent evaluation of toxicity-specific or forensic endpoints. No upgrading factor was applied. Publication bias could not be quantified. The qualitative judgment was that small discovery cohorts, extensive feature selection, and incomplete independent validation create reporting concerns, but do not establish non-publication of negative studies. The included corpus also contains multiplicity-adjusted null findings [82]. No additional publication-bias downgrade was applied.

The two remaining review questions are reported as structured narrative appraisals (Supplementary Table S3, Part B). The two primarily integrated studies support biological convergence, but do not demonstrate incremental forensic utility over conventional investigation. Confounding was handled inconsistently across the corpus, particularly for co-exposures, phenoconversion, ancestry, and postmortem conditions. These are statements about untested utility and methodological limitations, not GRADE-rated outcomes. Study-level directness remains distinct from both the narrative appraisals and outcome-level certainty.

## 4. Discussion

### 4.1. Principal Findings and Evidentiary Architecture

The evidence is weighted strongly toward PGx: 51 of 75 studies (68.0%) were assigned to that domain, compared with 17 (22.7%) across transcriptomic/epigenetic, metabolomic, proteomic, and integrated approaches; seven retained residual molecular classifications. The single proteomic investigation provides a preliminary study-level observation, not a replicated domain-level conclusion. Consequently, the framework integrates domains that differ markedly in volume, validation, and maturity. Across them, genotype–phenotype associations, metabolic ratios, tissue-distribution contrasts, and high-dimensional signatures address different inferential questions. Their effect magnitude, precision, replication, and proximity to fatal causation should be judged separately.

More than half the corpus required substantial extrapolation to individual postmortem interpretation (Figure 2). High directness does not imply analytical validation, diagnostic specificity, low risk of bias, or high GRADE certainty; a rigorous therapeutic study can remain indirect to fatal attribution. The evidence map therefore separates the clinical relevance of a molecular association from its demonstrated applicability to the forensic question.

Large estimates, broad molecular coverage, high internal AUCs, and direct fatal-outcome validation rarely coincided (Table 2; Supplementary Figure S1). Numerical abundance and apparent discrimination should not be interpreted as equivalent to evidentiary maturity.

### 4.2. Magnitude, Precision, and Realized Phenotype

The PGx findings illustrate why magnitude and precision require separate interpretation. Maternal CYP2D6 ultrarapid-metabolizer status had a large but unstable association with neonatal codeine toxicity (OR 16.50; 95% CI 1.09–250.18) [116], whereas the smaller association with poor pain control or opioid-related adverse reactions was more precisely estimated (OR 2.68; 95% CI 1.39–5.17) [47]. These different outcomes and populations cannot be combined into a single CYP2D6 effect.

This distinction is essential in molecular toxicology. A large estimate with an extremely wide interval may be less informative than a moderate, precise effect. Conversely, a non-significant association from a small genotype subgroup does not demonstrate absence of biological effect. Interpretation must separate direction, magnitude, interval width, allele frequency, sample size, validation, and mechanistic coherence.

Genotype also represents predicted functional capacity rather than enzyme activity at the relevant toxicological time. The realized phenotype results from interaction among inherited variation, dose, route, timing, age, ancestry, renal and hepatic function, pregnancy, chronic exposure, tolerance, co-medications, and enzyme inhibition or induction. Phenoconversion is therefore central. A genotype-predicted normal metabolizer may function as an intermediate or poor metabolizer during CYP2D6 inhibition, whereas inhibition of competing CYP3A4- or CYP2B6-dependent pathways may redirect metabolism toward CYP2D6-mediated bioactivation [46,48,61,74,95,96,101,108,113]. In forensic interpretation, genotype is a prior expectation that must be updated by measured concentrations, metabolite ratios, co-exposures, organ function, and the circumstances of death.

### 4.3. CYP2D6-Mediated Codeine and Tramadol Effects

This CYP2D6-codeine relationship predates the formal review window. Sindrup et al. linked the sparteine-oxidation phenotype to morphine formation and experimental codeine analgesia [33]; Gasche et al. described severe codeine intoxication in a patient with CYP2D6 duplication, interacting medication, and transient renal impairment [34]; and Kirchheiner et al. directly characterized CYP2D6 duplication and compared codeine/morphine pharmacokinetics after controlled codeine administration [35]. These publications provide the historical basis for interpreting the later evidence, without being treated as additional observations in the formal synthesis. The distinction between phenotype-based historical evidence and direct genetic characterization is retained; historical importance is not, by itself, a claim that every cited report meets the same molecular eligibility criterion.

Codeine provides a paradigmatic example of evidentiary convergence. The strongest evidence did not derive from a single genotype-death association, but from the combined interpretation of CYP2D6 genotype, dose, morphine formation, UGT2B7-mediated glucuronidation, ABCB1-mediated transport, interacting drugs, maternal exposure, and infant susceptibility [46,95,97,100,102,111,112,113,116,118]. Morphine-to-codeine ratios decreased across extensive-, intermediate-, and poor-metabolizer groups, but distributions overlapped substantially [46], precluding deterministic genotype-specific thresholds.

Controlled codeine administration showed that genotype alone correctly identified only 50.0% of subjects with extremely high morphine formation, whereas combined genotype and phenotyping identified 87.5% [102]. The resulting 37.5-percentage-point improvement demonstrates that realized metabolism is more informative than genotype considered in isolation.

The maternal–neonatal studies similarly showed that ultrarapid metabolism, UGT2B7 status, ABCB1 variation, maternal dose, and infant clinical depression jointly influenced toxicity [112,116]. Nevertheless, sparse extreme-genotype observations produced highly imprecise estimates. These findings support susceptibility assessment, not deterministic attribution.

Tramadol provided even stronger evidence that metabolic phenotype is dynamic. Postmortem ratios, hair-metabolite gradients, ultrarapid-metabolizer cases, sequencing studies, and pathway-based models consistently supported CYP2D6-dependent O-desmethyltramadol formation [48,56,60,64,87,90,91,101,119]. An N-desmethyltramadol/O-desmethyltramadol ratio above 7 separated all six poor metabolizers in one postmortem dataset [101], but external diagnostic performance was not assessed. Likewise, the monotonic reduction in O-desmethyltramadol from normal-function subjects to CYP2D6*10/10 and CYP2D6*5/*5 carriers was biologically coherent but based on small phenotype strata [87].

The machine-learning studies achieved apparent accuracies of up to 93.8–96.3% [90,91], yet genotype–phenotype discordance remained substantial, and genome-wide significant findings failed independent replication [76]. These results indicate derivation performance rather than proven transportability. Population structure, phenotype misclassification, feature-selection instability, environmental modifiers, and overfitting remain plausible explanations.

This distinction also helps define when postmortem pharmacogenetics is most likely to be useful: not as a routine molecular label attached to every opioid-positive death, but as a targeted adjunct when conventional toxicology reveals an otherwise unexplained parent/metabolite relationship, an unexpectedly high or low concentration, an atypical clinical course, or apparent discordance between administered dose and observed toxicity.

### 4.4. Methadone, Tissue Distribution, and Concentration Interpretation

Methadone showed a different but comparatively coherent PGx pattern. CYP2B6 variation was associated with reduced S-methadone clearance and with higher concentrations in several postmortem series [80,99,106,117]. Convergence across controlled PK and postmortem studies supports a genuine effect on disposition.

However, no genotype-specific lethal threshold can be derived. Methadone toxicity is modified by maintenance dose, tolerance, co-intoxicants, stereoselective metabolism, ancestry, cardiac susceptibility, survival interval, sampling site, and postmortem redistribution. Genotype may refine concentration interpretation, but cannot substitute for contextual toxicology.

The ABCB1 medulla-to-blood effects, with Cohen’s d values of 1.54–1.60, were among the largest standardized effects identified [79]. Their significance lies in demonstrating that target-organ distribution may vary despite similar peripheral concentrations. Femoral-blood methadone concentration may therefore incompletely represent cerebral exposure.

Nevertheless, brain or medulla concentrations are themselves influenced by redistribution, agonal circulation, tissue composition, sampling variability, and postmortem interval. ABCB1 findings should thus be interpreted as modifiers of the blood-to-target-organ relationship rather than as autonomous markers of causation. These findings broaden the forensic question from how much drug was present in blood to what biologically effective exposure may have reached the relevant target.

Evidence for CYP3A4-mediated methadone effects was less consistent. Selected variants were enriched in fatality subgroups, but genotype did not reliably predict concentration and control allele distributions were problematic [114]. This discrepancy illustrates the distinction between pathway plausibility and reproducible predictive utility.

### 4.5. Fentanyl, Sufentanil, and Contemporary Evidence Gaps

Fentanyl- and sufentanil-related studies identified potentially relevant CYP3A5- and CYP3A4-associated effects, including multiplicity-adjusted overdose associations, PK contrasts, and reduced adverse effects under genotype-guided dosing [63,67,71,85,109]. However, these studies evaluated non-fatal overdose, therapeutic PK endpoints, naloxone-treated toxicity, or obstetric analgesia rather than validated biomarkers of fatal intoxication.

The evidentiary distance from these endpoints to postmortem causal attribution is considerable. Fatal illicit exposure differs in dose, route, analogue identity, purity, polysubstance involvement, tolerance, and survival bias. A PGx effect identified in therapeutic or non-fatal settings cannot be directly extrapolated to lethal fentanyl intoxication.

**Figure 4:**
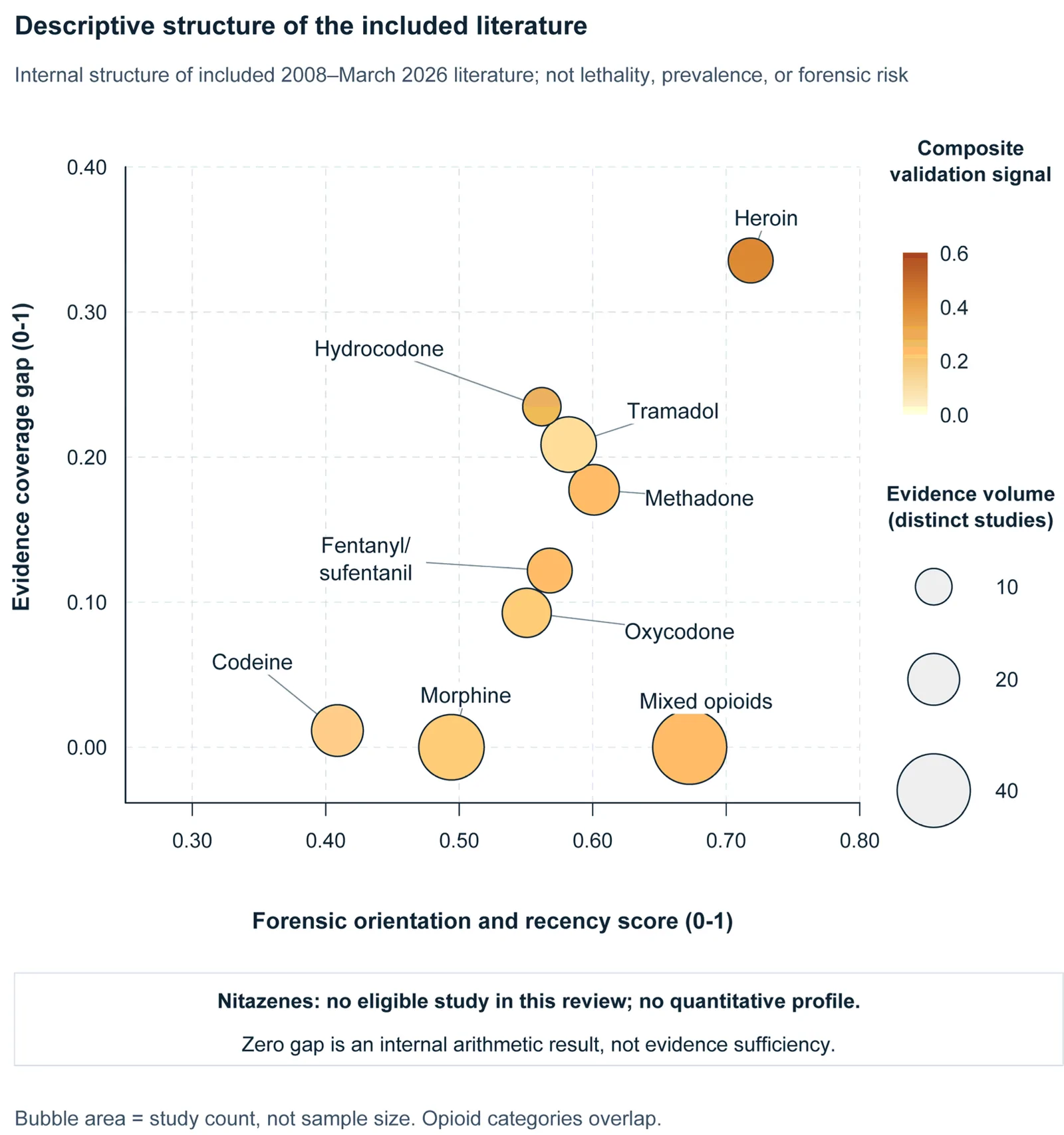
Descriptive forensic orientation, recency, and evidence coverage within the included literature. Horizontal position is the forensic orientation and recency score *F* = (*B* + *D* + *R*)/3; vertical position is the coverage gap *G* = max(0, *F − S*), where evidence support *S* = (*V + M + C*)/3. *B* is the normalized forensic-setting representation component; *D*, directness; *R*, recency; *V*, study count; *M*, molecular breadth; and *C*, the composite validation signal. Bubble area represents distinct studies and color represents *C*. Heroin’s higher *F* than fentanyl/sufentanil (0.718 versus 0.568) reflects greater forensic-event representation and directness, despite lower recency. Both contain 15 studies. Its larger *G* (0.336 versus 0.122) combines higher *F* with lower *S*, reflecting narrower molecular breadth despite a higher *C*. These differences do not rank toxicological importance, lethality, prevalence, or forensic risk. A zero gap does not establish evidence sufficiency. Categories overlap; nitazenes receive no quantitative profile. Formulas and component values are provided in Supplementary Methods S1.

Figure 4 describes the composition of the included literature, independently of contemporary mortality or toxic potency. The heroin–fentanyl/sufentanil contrast is explained by the component values in its caption and Supplementary Methods S1. Nitazenes receive no quantitative score. Their empty eligible category is specific to this review’s human PGx/endogenous-omics criteria and must not be read as an absence of nitazene research: the search retrieved drug-metabolism, analytical-toxicology, and other records that did not enter the eligible molecular synthesis (Supplementary Table S4).

### 4.6. Multi-Omic Evidence and Forensic Specificity

Downstream omics can discriminate sampled groups while remaining biologically or forensically non-specific. Internally cross-validated epigenetic and network models showed high apparent discrimination, but their matrices, endpoints, preprocessing, and validation differed [53,65,68,72]. They cannot be interpreted as a common diagnostic accuracy for opioid-related death.

Postmortem preservation also determines whether molecular signatures are measurable and comparable. Human tissue RNA profiles change with postmortem interval through both degradation and tissue-specific transcriptional responses, requiring consideration of RNA integrity and collection conditions [130]. DNA methylation can be relatively stable under controlled conditions, but stability differs across loci, tissues, and assays. Experimental brain studies found relative preservation of global cytosine modifications while some histone acetylation marks were more labile [131]; deteriorating DNA integrity can increase variability in bisulfite-based methylation measurements [132]. Human skeletal-muscle studies likewise show target-dependent protein degradation related to elapsed time and accumulated temperature exposure [133]. These observations support documenting postmortem interval, temperature and refrigeration history, sampling site, preservation delay, storage conditions, and analyte-specific quality metrics, with comparable handling of cases and controls. They provide no universal acceptable postmortem interval and do not directly validate opioid-specific brain signatures. Agonal and postmortem changes must therefore be distinguished from exposure-related biology within each tissue and platform.

Internal cross-validation estimates performance within the sampled data structure but does not establish transportability across populations, tissues, laboratories, analytical platforms, postmortem intervals, or alternative causes of death. In high-dimensional studies, optimistic performance may result from inadequate nesting of feature selection, class imbalance, batch effects, small effective sample size, or model-selection bias.

The externally validated oxycodone metabolomic model retained 80% sensitivity but only 74% specificity [70]. Approximately one quarter of controls were therefore classified as positive under validation conditions. Such performance may support an integrated forensic assessment, but is insufficient for autonomous attribution. Acylcarnitine perturbations may reflect mitochondrial dysfunction, hypoxia, critical illness, or terminal stress rather than opioid-specific injury.

The fentanyl urinary profiles classified exposure-associated patterns, not fatal causation [78]. Likewise, clustering of a fatal infant methadone case with fatal asphyxia cases illustrates detection of terminal hypoxic biology without identification of the initiating agent [59].

Despite these limitations, pathway-level convergence was substantial. OPRM1 variation was linked to methylation, mRNA expression, receptor density, and signaling [107]. Brain and blood miRNA networks shared enriched targets [58], methylation-expression analysis identified 272 overlapping genes [86], and H3K27ac, ELK1, glutamatergic, GABAergic, inflammatory, neurovascular, synaptic, and mitochondrial pathways recurred across studies [53,54,57,58,65,69,77,82,86,88,98,107]. This strengthens biological plausibility but does not establish individual calibration.

Null findings remain equally important. No CpG survived FDR correction in acute intoxication [82]; OPRM1 and ABCB1 did not predict tramadol seizures [49]; CYP2D6 did not consistently predict absolute tramadol concentrations [55]; CYP3A4 did not reliably predict methadone levels [114]; and selected OPRD1, DRD2/ANKK1, and ABCB1 associations failed replication or multiplicity correction [52,109]. These findings constrain overgeneralization and confirm phenotype specificity.

### 4.7. Molecular-Forensic Integration and Implications

Molecular integration should connect inherited functional potential with measured parent-drug and metabolite patterns, tissue distribution, co-exposures, and dynamic modifiers. Concordance among these observations may support an explanation for an atypical toxicological phenotype; discordance should prompt review of timing, phenoconversion, analytical factors, and alternative causes.

Downstream omics can contribute information about biological response, but evidence of a response is not evidence that an opioid initiated the fatal process. A useful integrated model must test whether adding molecular information improves a defined outcome beyond conventional toxicology and the medicolegal investigation.

The framework therefore treats molecular findings as explanatory modifiers of causal inference. No study-derived score supplies a numerical probability that an opioid caused an individual death, and no absence of an unvalidated signature excludes opioid toxicity.

Future studies should integrate genotype, copy-number variation, phenoconversion, dose, timing, organ function, co-medications, metabolic ratios, and the realized toxicological phenotype within the same model. Postmortem investigations require standardized sampling, explicit survival and postmortem intervals, alternative-cause controls, and systematic assessment of tolerance, redistribution, and co-intoxicants. Case definitions must distinguish exposure, intoxication, contributory drug involvement, and death caused primarily by the opioid; a model designed to recognize exposure answers a fundamentally different question from a model intended to discriminate fatal intoxication from incidental detection. Omics models require prespecified pipelines, fully nested validation, calibration, locked feature selection, assessment of postmortem stability, and independent replication across laboratories and forensic populations. Such studies should test whether molecular information adds incremental value beyond conventional variables rather than merely showing an association with opioid exposure.

**Figure 5:**
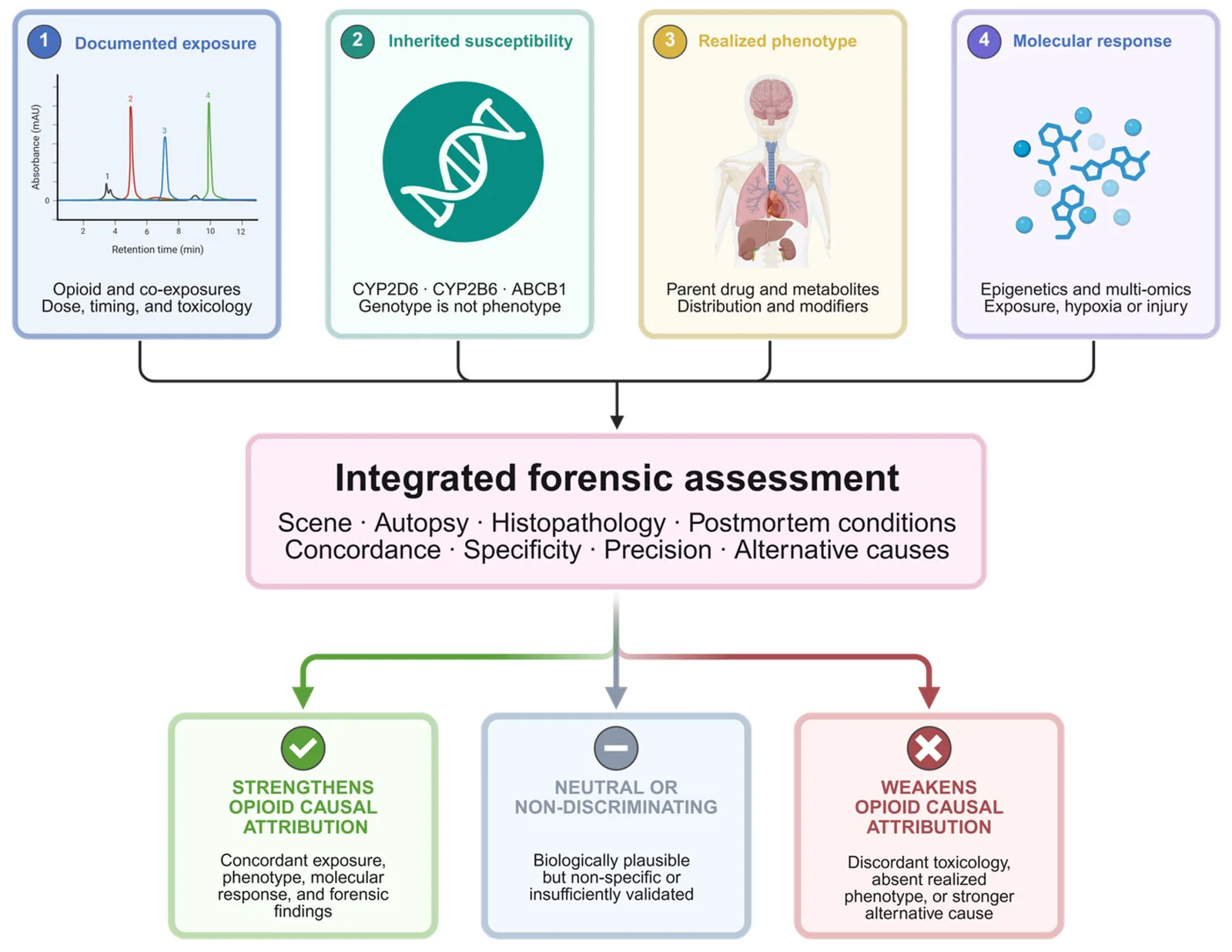
Simplified framework for integrating molecular findings into forensic assessment of opioid-related deaths. Documented exposure, inherited susceptibility, the realized pharmacokinetic/pharmacodynamic (PK/PD) phenotype and downstream molecular responses are assessed jointly with the complete forensic investigation. Interpretation considers concordance, specificity, directness, precision, replication and alternative causes. Integrated findings may strengthen attribution, remain neutral or non-discriminating, or weaken attribution; absence of an unvalidated molecular signature alone cannot exclude opioid toxicity. Arrows indicate integration of evidence, not a validated diagnostic sequence or quantitative probability of causation. No eligible nitazene-specific molecular studies were identified within the review’s search scope and period. Created in BioRender. D’Anna, T. (2026) https://BioRender.com/ghe52d9.

Figure 5 constitutes the main take-home message of the review. It should be interpreted as an evidentiary-convergence framework rather than a diagnostic algorithm. Molecular findings may strengthen opioid attribution when they are exposure concordant, biologically coherent, sufficiently precise, independently replicated, and consistent with toxicology, scene investigation, autopsy, histopathology, postmortem conditions, and exclusion of alternative causes. They may remain neutral when non-specific or insufficiently validated, and may weaken attribution when discordant with the expected toxicological phenotype. Molecular evidence should therefore refine and calibrate conventional forensic causal assessment, but cannot replace it.

### 4.8. Certainty of Evidence, Strengths, and Limitations

GRADE certainty was very low for all four outcome-focused bodies. This does not negate biological plausibility; it indicates uncertainty in the magnitude, specificity, reproducibility, and forensic transferability of the findings. Integration utility remains untested, and confounder control is a methodological appraisal rather than a separate GRADE outcome. High study-level directness should not be conflated with high certainty.

Strengths include explicit eligibility criteria, preservation of original effect metrics, design-specific JBI appraisal, and study-level evidence mapping. Limitations include heterogeneity that precluded defensible pooling, dependence among estimates from the same study, small or selected samples, incomplete confounder reporting, and scarce independent validation. The orientation, evidence-support, and coverage-gap scores are internally normalized descriptive constructs whose coding and weights have not been validated against clinical or forensic outcomes. Their numerical order depends on this selected literature corpus.

Embase was not searched, and its pharmacological and pharmacogenetic coverage may include reports not indexed in the three databases used. Eligibility was restricted to English-language, peer-reviewed publications; grey literature, unpublished studies, and non-English reports were excluded. These restrictions may introduce language and dissemination bias and disproportionately affect sparse domains, including emerging synthetic opioids. Their direction and magnitude cannot be quantified from the included studies. The identified evidence gaps consequently apply to this search strategy and eligibility scope, not to all research activity.

The common publication window does not capture the complete historical evidence for established drug–gene relationships [33,34,35], and the internally normalized scores should not be extrapolated to an all-period evidence base. The supplementary audit now documents ten pre-2008 reports retained under the stated PEOS interpretation among 302 deduplicated records screened (Supplementary Table S5), but it does not establish the total size of the excluded historical literature. The original strategy required a toxicity, overdose, postmortem, cause-of-death, or forensic concept in addition to opioid and molecular concepts. That retrieval restriction is narrower than the PEOS allowance for opioid metabolism, transport, and response and can miss pharmacokinetic or analgesic studies without the required searchable terms. The non-retrieval of the cited Sindrup and Kirchheiner reports illustrates this limitation. Search-term coverage was also incomplete for genotype, gene-duplication, and metabolizer terminology. These sensitivity limitations are not confined to pre-2008 publications and may also affect capture of otherwise PEOS-compatible studies within the formal period. The historical audit measures the yield of the unchanged strategy; it does not remedy its incomplete coverage or quantify the effect of missing studies on the synthesis.

The independently assigned directness categories were resolved by discussion, but no pre-consensus agreement statistic is available. Finally, protocol archiving followed full-text selection; it documents the protocol publicly without constituting prospective preregistration.

## 5. Conclusions

This time-bounded systematic review identifies a predominantly pharmacogenetic evidence base, with the most coherent findings concerning CYP2D6-dependent codeine and tramadol bioactivation, CYP2B6-dependent methadone disposition, and selected tissue-distribution effects. The much smaller omics literature provides biological hypotheses and exposure-associated signatures with limited independent validation and fatal-outcome specificity. Certainty is very low across the four outcome-focused bodies appraised with GRADE; incremental forensic utility has not been demonstrated. Molecular information is most useful when it helps explain the relationship between inherited susceptibility, the measured toxicological phenotype, and the observed clinical or fatal outcome. Application requires corroboration from toxicology, scene investigation, autopsy, histopathology, and assessment of alternative causes. Future studies should evaluate externally validated, opioid-specific models and their added value over conventional investigation.

## Figures and Tables

**Table 1:** Concise overview of the 75 included studies by molecular evidence domain.

Evidence Domain and Study Types, *n* (%)	Main Molecular Targets	Opioids Represented	Principal Findings	Forensic Relevance and Limits
Pharmacogenetics/pharmacogenomics, 51 (68.0%) Clinical, experimental and postmortem studies	CYP2D6, CYP2B6, CYP3A4/5; UGT2B7, ABCB1, OPRM1, and COMT	Codeine, tramadol, morphine, methadone, oxycodone, hydrocodone, fentanyl/sufentanil, buprenorphine; mixed opioids	Genotype associations with bioactivation, clearance, metabolic ratios, tissue distribution and adverse effects; effects vary by drug and endpoint [56,79,93,99].	Can contextualize susceptibility and toxicological findings. Genotype alone does not establish realized phenotype, toxicity or cause of death.
Transcriptomic/epigenetic markers, 11 (14.7%) Postmortem, translational and placental studies	H3K27ac, DNA methylation/hydroxymethylation, OPRM1, miRNAs, and regulatory pathways	Heroin, morphine and mixed opioids, including prenatal exposure	Altered chromatin, methylation, and gene expression; strong internal discrimination in selected datasets [53,54,57,68,72,82].	Supports biological plausibility. Acute toxicity must be distinguished from chronic exposure, developmental effects, and terminal physiology; external validation is limited.
Metabolomic and preliminary proteomic evidence, 4 (5.3%) 3 metabolomic studies; 1 postmortem proteomic study	Acylcarnitines and broader metabolic profiles; region-specific neural proteins	Oxycodone, methadone, fentanyl, and heroin	Metabolomic exposure/intoxication profiles [59,70,78]; one unvalidated heroin-associated brain proteomic observation [69].	Metabolic patterns may reflect hypoxia or other exposures. The single proteomic study supports hypotheses, without a replicated signature or validated forensic classifier.
Integrated multi-omic analyses, 2 (2.7%) Cross-tissue and network analyses	miRNA-mRNA networks and convergent neurobiological gene networks	Mixed opioids; heroin, fentanyl, hydrocodone, and oxycodone	Cross-tissue regulatory changes and convergent OUD-related networks; validation was cross-tissue or internal [58,65].	Generates mechanistic hypotheses. Demonstrated added value for individual forensic causal attribution is lacking.
Other/unclassified molecular evidence, 7 (9.3%) Forensic case reports, series and case–control studies	CYP2D6, CYP2B6, CYP2C19, ABCB1, OPRM1, COMT, and other candidate variants	Methadone, codeine/morphine, heroin, and hydrocodone	Genetic associations, phenoconversion, and drug interactions contextualized discordant metabolism or fatal toxicity [74,100,113].	Case-level explanatory evidence with heterogeneous methods; this residual coding category includes targeted PGx reports.

Counts retain the mutually exclusive primary-domain coding of the original extraction. Metabolomics (3 studies) and the single proteomic investigation are combined here only for concise presentation; the total remains 75. Integrated approaches remain in their assigned primary category. The residual category includes targeted PGx reports not classified as PGx by the original coding scheme. Study types and opioid lists are descriptive. Complete study-level characteristics and risk-of-bias assessments are provided in Supplementary Table S2. PGx, pharmacogenetics/pharmacogenomics; OUD, opioid use disorder; miRNA, microRNA; mRNA, messenger RNA.

**Table 2:** Quantitative architecture of the effect-size-oriented synthesis.

Component	Numerical Result	Statistical Interpretation
Included studies	75	Primary study-level analytical units
Initial numerical occurrences	606	Audit-level extraction; repeated reporting permitted
Deduplicated estimands	332	Unique by study, metric, value, unit, and CI
Substantive estimands	124	Isolated significance-only records excluded
Studies with ≥1 numerical occurrence	66/75 (88.0%)	Numerical reporting was common but not universal
Studies displayed in Supplementary Figure S1	43/75 (57.3%)	Studies meeting display-level quantitative requirements
Estimands displayed in Supplementary Figure S1	114/124 (91.9%)	Manuscript-facing study-level numerical estimands
Supplementary Figure S1 validation tiers, D/IV/CV/EV	29/3/5/6	Highest validation level assigned per displayed study
PGx studies	51/75 (68.0%)	Dominant evidence domain
Transcriptomic/epigenetic studies	11/75 (14.7%)	Heterogeneous tissues, endpoints, and platforms
Metabolomic studies	3/75 (4.0%)	Small but comparatively direct toxicological domain
Proteomic studies	1/75 (1.3%)	No independently validated diagnostic classifier
Integrated multi-omic studies	2/75 (2.7%)	High-dimensional and predominantly internally validated
Low/moderate/high risk of bias	36/36/3	No study excluded solely on methodological score
High/moderate forensic interpretative directness	25/11	36/75 studies, 48.0%
Low/no forensic interpretative directness	31/8	39/75 studies, 52.0%
Acute overdose or fatal-toxicity subgroup	37/75 (49.3%)	Most direct toxicity subgroup
Chronic-toxicity/OUD subgroup	4/75 (5.3%)	Studies coded as chronic toxicity/OUD in the toxicity-timing classification
Other or unclear toxicity timing	34/75 (45.3%)	Residual timing category; completes the 75-study partition
Classical-opioid subgroup	57/75 (76.0%)	Evidence concentrated on established opioids
Fentanyl/sufentanil subgroup	15/75 (20.0%)	Few directly validated fatality biomarkers
Mixed or unspecified opioids	3/75 (4.0%)	High exposure heterogeneity

Note: The toxicity-timing categories are mutually exclusive and exhaustive: acute overdose/fatal toxicity (37), chronic toxicity/OUD (4), and other or unclear timing (34). They differ from the exposure-context categories because chronic exposure may coexist with an acute fatal endpoint. Supplementary Figure S1 displays the structured quantitative subset; Figure 3 is the molecular-opioid count matrix. Abbreviations: CI, confidence interval; CV, cross-validation; D, discovery/descriptive analysis; EV, external/independent validation; IV, internal/technical validation; OUD, opioid use disorder; PGx, pharmacogenetics/pharmacogenomics.

**Table 3:** Principal CYP2D6- and tramadol-related quantitative findings.

Study	Context	Numerical Effect	Precision or Statistical Evidence	Technical Interpretation
Naruge et al. [45]	Therapeutic tramadol	Pain change −1.50 IM vs. −0.67 EM; discontinuation 33% vs. 16%	Underpowered; no formal genotype effect	Numerical efficacy-toxicity gradient only
Frost et al. [46]	Codeine deaths	Mean M/C 0.058 EM, 0.043 IM, 0.025 PM	R^2^ = 0.21 for codeine-morphine relationship	Directional phenotype gradient with extensive overlap
St Sauver et al. [47]	Therapeutic mixed opioids	OR 2.68 for poor pain control or adverse reaction	95% CI, 1.39–5.17; *p* = 0.003	Clinically interpretable phenotype association, indirect for fatality
Elkalioubie et al. [48]	Near-fatal tramadol	MR1 2.54; MR2 11.4; tramadol 3.22 mg/L	Single case	Mechanistically coherent UM plus drug-interaction effect
Wendt et al. [76]	GWAS replication	Five FDR-significant loci	Adjusted *p* = 1.70 × 10^−11^ to 5.48 × 10^−9^; none replicated	Strong discovery signal without external reproducibility
Arafa and Atteia [84]	Chronic tramadol	Grade ≥ 2 hepatotoxicity 42.9% vs. 0%; ALT-M1 r = 0.97	*p* = 0.0049; correlation *p* < 0.0001	Large biological effect requiring independent confirmation
Yu et al. [87]	Tramadol in hair	ODMT/tramadol 0.13, 0.04, and 0.02 across decreasing CYP2D6 activity	*p* > 0.05 because of small strata	Monotonic but imprecise genotype gradient
Wendt et al. [90]	Postmortem pathway model	h^2^ = 0.821; R^2^ = 0.329; accuracy ≤ 93.8%	Bonferroni correction; pathway vs. CYP2D6 *p* = 0.019	Strong apparent multilocus signal
Wendt et al. [91]	Postmortem machine learning	Random forest accuracy 96.3%; RMLR 52.3%	Discordance *p* = 1.57 × 10^−31^	Performance depended strongly on representation and model
Fonseca et al. [101]	Postmortem tramadol	NDT/ODT > 7 separated all six PM cases	Mann–Whitney *p* < 0.05	Strong within-dataset phenotype discrimination
Lötsch et al. [102]	Controlled codeine	High-formation classification 50.0% genotype, 62.5% phenotype, 87.5% combined	Descriptive classification	Integrated genotype–phenotype classification improved by 37.5 percentage points
Madadi et al. [112]	Breastfeeding codeine	OR 8.40; maternal dose 59% higher	95% CI, 4.70–47.00; *p* ≤ 0.001; dose *p* = 0.004	Large combined genetic-exposure effect with limited precision
Lam et al. [113]	Codeine deaths	Morphine 0 vs. 17 ng/mL by ABCB1 group	*p* = 0.004; inhibitor gradient *p* = 0.0011	Transport and phenoconversion materially affected toxicology
Sistonen et al. [116]	Maternal-infant codeine	ABCB1 OR 6.21; CYP2D6 UM OR 16.50; multilocus OR 2.68	AUC 0.87; 95% CI, 0.75–0.98; sensitivity 80%; specificity 87%	High internal discrimination; sparse extreme-genotype data
Stamer et al. [119]	Tramadol respiratory depression	(+)-ODT approximately 2-fold and 3-fold higher	Single case; complete naloxone reversal	UM status interacted with renal impairment

Abbreviations: ALT, alanine aminotransferase; AUC, area under the receiver operating characteristic curve; CI, confidence interval; EM, extensive metabolizer; FDR, false discovery rate; GWAS, genome-wide association study; h^2^, heritability estimate; IM, intermediate metabolizer; M/C, morphine-to-codeine ratio; M1, O-desmethyltramadol; MR1, tramadol-to-O-desmethyltramadol metabolic ratio; MR2, tramadol-to-N-desmethyltramadol metabolic ratio; OR, odds ratio; PM, poor metabolizer; R^2^, coefficient of determination; RMLR, regularized multinomial logistic regression; UM, ultrarapid metabolizer.

**Table 4:** Pharmacokinetic, tissue-distribution, and pharmacodynamic effects beyond CYP2D6.

Study	Axis	Effect Estimate	Statistical Precision	Main Inferential Limitation
Wong et al. [50]	COMT/OPRM1	Sickness aOR 7.10; nausea aOR 5.47	Wide CIs; *p* = 0.01–0.02	Small cancer cohort
Shu et al. [63]	CYP3A4-guided sufentanil	Dose 87.6%; vomiting −6.31 points; retention −10.16 points	Pain-equivalence *p* > 0.70; adverse effects *p* < 0.05	Obstetric therapeutic setting
Lambert et al. [67]	CYP3A5/DRD2/NK1R	OR 6.96, 5.58, and 0.28	Bonferroni-adjusted; CIs exclude 1	Self-reported non-fatal overdose
Iwersen-Bergmann et al. [79]	ABCB1 transport	Medulla/blood *d* = 1.54–1.60	95% CIs 1.14–2.05 and 1.13–2.29	High co-intoxicant prevalence
Ahmad et al. [80]	CYP2B6-methadone	Methadone 1.67 ± 0.85 vs. 0.59 ± 0.05 mg/L	*p* = 0.002	Postmortem dose/tolerance uncertainty
Talal et al. [81]	CYP2B6	S-EDDP/methadone 1.342-fold lower	*p* = 0.012	Stable-treatment pharmacokinetics
Chou and Hsu [83]	OPRM1	Female GG-AA difference +7.9 mg at 24 h; +16.4 mg at 48 h	*p* = 0.04	Sex-specific subgroup
Rowsell et al. [94]	OPRM1 respiratory response	HCVR −0.90 in AG; genotype interaction *p* = 0.014	95% CI, −1.46 to −0.33	Null overall primary outcome
Kharasch et al. [99]	CYP2B6-methadone	S-clearance −35% (*1/*6), −45% (*6/*6); *4 clearance 3–4-fold higher	Clearance-metabolism *r* = 0.57 and 0.82; *p* < 0.001	Controlled PK, not fatality risk
Kolesnikov et al. [103]	OPRM1/COMT	Morphine −19.7 mg	*p* < 0.05	Combined-genotype subgroup
Bunten et al. [106]	CYP2B6/OPRM1	Methadone 0.93 vs. 0.56 mg/L; benzodiazepines 2.4-fold higher	*p* = 0.041 and *p* = 0.004	Co-intoxicant dependence
Bastami et al. [115]	UGT2B7/OPRM1/ABCB1	Morphine 12 ± 9 vs. 37 ± 22 mg; model R^2^ ≈ 0.30	*p* = 0.005	Small perioperative cohort

Abbreviations: aOR, adjusted odds ratio; CI, confidence interval; HCVR, hypercapnic ventilatory response; OR, odds ratio; PK, pharmacokinetics; R^2^, coefficient of determination.

**Table 5:** Principal pharmacogenetic findings and their forensic interpretative relevance.

Evidence Domain	Principal Opioid(s)	Molecular Determinant	Most Consistent Observation	Forensic Interpretation
Metabolic PGx	Codeine; tramadol	CYP2D6	Active-metabolite formation and metabolic ratios were more consistently related to phenotype than absolute parent-drug concentrations.	Interpret with measured parent/metabolite pattern and interacting drugs; genotype alone is insufficient.
Metabolic PGx	Methadone	CYP2B6	Reduced-function alleles were associated with altered stereoselective clearance, metabolic ratios, or concentrations.	May explain atypical disposition when concordant with case toxicology.
Transport PGx	Methadone	ABCB1	Association with medulla-to-blood distribution in a postmortem study.	Potential target-site relevance; independent replication required.
Pharmacodynamic PGx	Multiple opioids	OPRM1; COMT	Associations with dose requirement, adverse effects, or ventilatory response were heterogeneous.	Currently supportive at most; limited direct causal value.
Emerging opioids	Fentanyl/sufentanil; nitazenes	Candidate metabolic pathways	Sparse fentanyl-specific evidence; no eligible nitazene-specific study.	Major evidence gap; avoid extrapolation from older opioids.

Note: The table provides a qualitative interpretative summary of the included evidence. Abbreviations: PGx, pharmacogenetic/pharmacogenomic.

**Table 6:** Quantitative omics findings.

Study	Domain and Matrix	Molecular Magnitude	Performance or Validation	Interpretative Weight
Corradin et al. [53]	H3K27ac, postmortem brain	388 hypoacetylated peaks; seven-feature model	AUC 0.972 ± 0.037, five-fold CV	Excellent internal discrimination; no external forensic validation
Rompala et al. [54]	5mC/5hmC, orbitofrontal cortex	397 5mC and 1740 5hmC CpGs	GWAS enrichment OR 1.876; R^2^ = 0.66 external-reference agreement	Stronger 5hmC than 5mC convergence
Mendez et al. [57]	iPSC-neuron transcriptome	312 DE genes; 20 overlap, 11 concordant	Maturity correlation *r* = 0.69	Partial cross-dataset validation; FDR < 0.20
Grimm et al. [58]	Brain/blood miRNA-mRNA	402 genes; 89 brain and 104 blood miRNAs; 79 shared targets	Overlap *p* = 1.92 × 10^−20^; brain-blood *r* = −0.46	Network convergence stronger than direct surrogate correlation
Chighine et al. [59]	Urinary NMR, fatal infant methadone	First 3 PCs 63.3% variance	Qualitative clustering only	Hypoxic-response signal, not methadone-specific
Sullivan et al. [65]	Integrated multi-omic network	211 genes, 50 high-confidence, 48 druggable	Cross-validated AUC 0.94	Strong mechanistic integration; not a clinical fatality test
Radhakrishna et al. [68]	Placental methylome	111 CpGs; differences −10.62% to +10.40%	AUC 0.57–0.92	High exposure/NOWS discrimination, low adult-forensic directness
Sürmen et al. [69]	Brain proteome	288 proteins at ≥2-fold, FDR ≤ 0.01	No classifier	Mechanistic neurotoxicity only
Elmsjö et al. [70]	Postmortem blood metabolomics	25 acylcarnitines, FC 0.46–0.79	R^2^ = 0.41; Q^2^ = 0.21; external Se/Sp 80%/74%	Most directly externally tested fatality omics signal
Radhakrishna et al. [72]	Placental transporter methylome	69 SLC, 11 ABC, 7 PKC genes	AUC 0.84–0.89	Internally strong, clinically indirect
Wang et al. [77]	Plasma exosomal miRNA	Four significant miRNAs	*p* < 0.001–0.010	Heroin–HIV interaction limits specificity
Amante et al. [78]	Urinary fentanyl metabolomics	186-variable SIMCA model	CV Se 96–100%; Sp 74–76%	Strong exposure detection, not fatality attribution
Shu et al. [82]	Brain methylome	No CpG survived FDR	PhenoAge β = 2.24, SE = 1.11, *p* = 0.045	Largely negative genome-wide result
Liu et al. [86]	Brain methylome/transcriptome	272 overlapping genes	Overlap *p* = 8.42 × 10^−21^	Cross-omic biological convergence
Egervari et al. [88]	Histone acetylation/transcriptomics	Human *r* up to 0.70; animal reductions 36–53%	Human-animal validation	Mechanistic chronic-exposure evidence
Daws et al. [98]	ELK1/OPRM1 network	ELK1 133%; 1518 genes	R^2^ up to 0.70 overall and 0.95 in small subgroup	Strong signal with sparse-subgroup instability
Oertel et al. [107]	OPRM1 genotype-methylation	mRNA −50%; signaling −60%	Interaction *p* =0.002 and 0.019	Direct genetic–epigenetic–functional convergence

**Table 7:** Interpretative value and principal limitations of the multi-omic evidence identified in the review.

Omics Layer	Representative Signal	Potential Contribution	Principal Limitation	Current Status
Metabolomics	Acylcarnitine and broader metabolic perturbations; exposure classifiers	May capture altered energy metabolism, hypoxia, or systemic response.	Limited etiologic specificity; exposure classification is not fatal-causation classification.	Promising, exploratory
Epigenomics	Differential methylation/hydroxymethylation and histone-acetylation regions	May reflect chronic exposure, neuroadaptation, or regulatory response.	Tissue specificity, chronic-use confounding, postmortem effects, limited external validation.	Exploratory
Transcriptomics	Differential gene/miRNA expression	May identify synaptic, immune, hypoxic, or stress pathways.	Agonal/postmortem confounding and limited brain-to-blood correspondence.	Exploratory
Single proteomic investigation	Region-specific differential protein expression	Preliminary mechanistic observation	One study; no independent replication or validated diagnostic performance.	Single-study evidence
Integrated multi-omics	Cross-platform networks and high-dimensional classifiers	May improve mechanistic integration and target discovery.	Overfitting risk; endpoints often concern OUD/exposure rather than cause of death.	Research-stage

Note: The table provides a qualitative interpretative summary of the included evidence. Abbreviations: OUD, opioid use disorder.

## Data Availability

No new data were created or analyzed in this study. Data sharing is not applicable to this article.

## Supplementary Materials

The following supporting information can be downloaded at https://www.mdpi.com/article/10.3390/ijms27188332/s1.
