# Supplementary material for: Pharmacogenetic Evidence in Opioid-Related Toxicity and Death with an Appraisal of Emerging Multi-Omics Studies: A Systematic Review
Source: Int J Mol Sci. 2026 Sep 19;27(18):8332. doi: 10.3390/ijms27188332 (PMC13607749; doi:10.3390/ijms27188332)
Supplement: Supplementary file 1 [file ijms-27-08332-s001.zip › FigureS1_Quantitative_Molecular_Evidence.pdf]

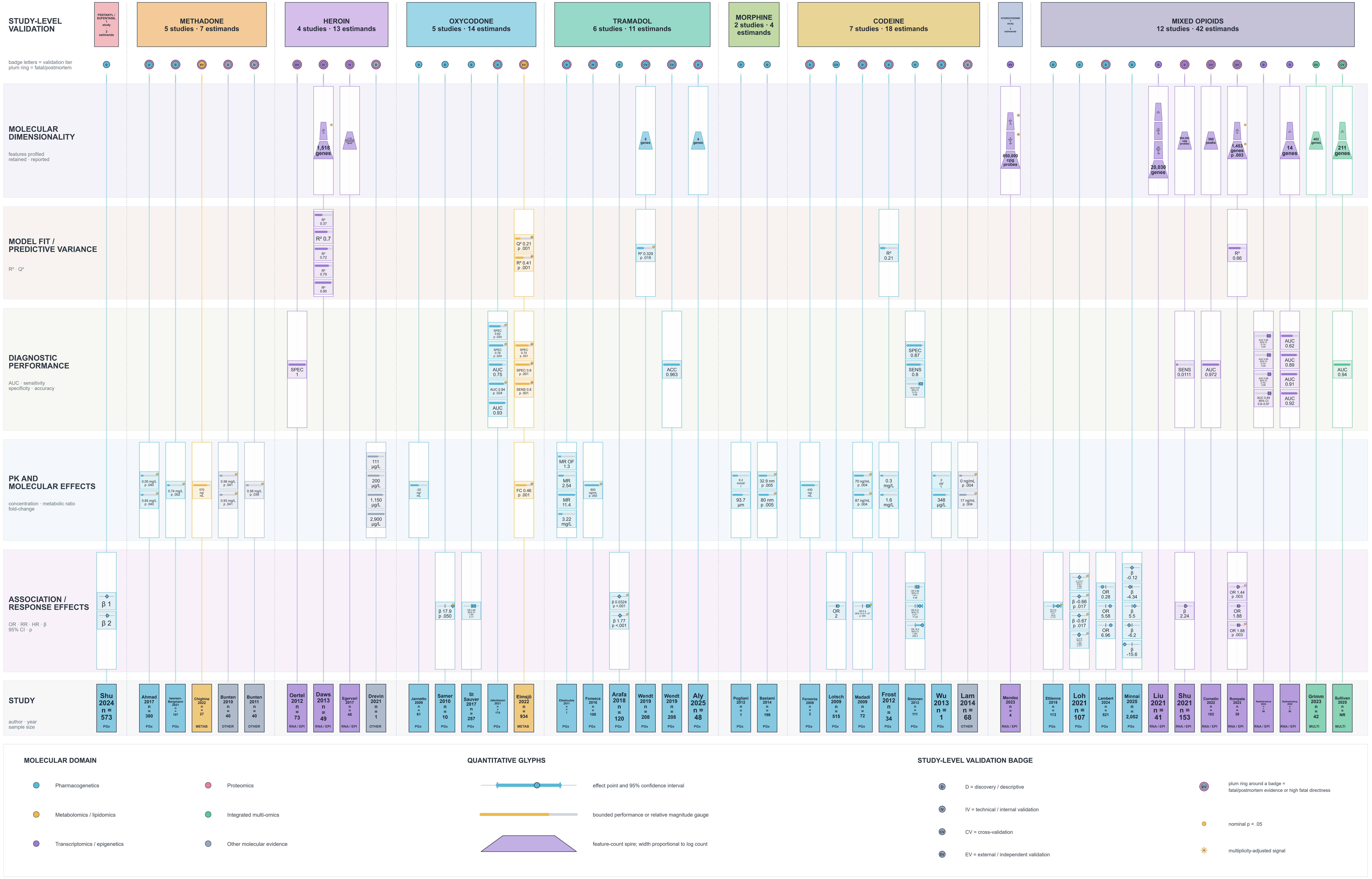

Supplementary Figure S1. Quantitative architecture of the molecular evidence. The 114 displayed estimands from 43 studies were concentrated in mixed -opioid, codeine, oxycodone, heroin, and tramadol evidence. Large association effects, extensive molecular dimensionality, high apparent discrimination, external validation, and direct relevance to fatal or postmortem outcomes rarely co-occurred within the same studies; most displayed evidence remained discovery-level or internally validated.
