# Supplementary material for: Pharmacogenetic Evidence in Opioid-Related Toxicity and Death with an Appraisal of Emerging Multi-Omics Studies: A Systematic Review
Source: Int J Mol Sci. 2026 Sep 19;27(18):8332. doi: 10.3390/ijms27188332 (PMC13607749; doi:10.3390/ijms27188332)
Supplement: Supplementary file 1 [file ijms-27-08332-s001.zip › MethodsS1_Figures2_4.pdf]

Supplementary Methods S1. Detailed computational methods and audit framework for maint-text Figures 2-4.

1. Purpose, analytical status, and unit of analysis

Figures 2-4 provide descriptive quantitative maps of the structure, molecular content, forensic orientation, and relative evidence coverage gaps of the 75 studies included in the systematic review. Each figure is presented separately to improve readability. The unit of analysis in Figure 2 is the individual study. Figures 3 and 4 use deterministic study-opioid assignments; consequently, a study may contribute to more than one opioid category when more than one opioid is explicitly identified in the corresponding extraction fields. Repeated occurrences of the same study within the same analytical relation are removed before aggregation.

The analyses underlying Figures 2-4 constitute descriptive quantitative aggregation and visual evidence mapping. The analytical script exports study-level mappings, recoding decisions, molecular links, component scores, and final opioid-level values as audit files. All profiles describe the internal structure of the 75 eligible studies published from 2008 to March 2026. Scores and indices are exploratory descriptive constructs, not validated epidemiological or forensic assessment tools; they do not measure lethality, prevalence, or forensic risk. They have not been calibrated to external mortality data, toxicity outcomes, or individual cause-of-death adjudication.

2. Main-text Figure 2 – Study-level distribution of the included evidence

2.1. Structure

Each of the 75 included studies contributed one and only one alluvial pathway through four mutually exclusive study-level dimensions: principal evidence category, opioid class, principal outcome domain, and forensic interpretative directness. Each ribbon represents one study; bundled ribbon widths encode study counts. A case report and a large cohort each contribute one pathway: the figure is not weighted by participant numbers, precision, study design, or evidential strength. Very small nodes include additional vertical padding to keep every category label legible.

2.2. Coding rules

Dimension	Categories	Interpretation
Principal evidence category	Clinical PGx; forensic toxicology; postmortem genetics; epigenetic; metabolomic; proteomic; integrated; other	Mutually exclusive principal evidentiary domain assigned during extraction
Opioid class	Mixed opioids; natural opiates; semisynthetic; synthetic/fentanyl; methadone; tramadol	Mutually exclusive exposure class used for the study-level river
Principal outcome domain	Clinical toxicity; forensic/postmortem; molecular signature; pharmacological response; toxicological interpretation; neonatal/perinatal	Mutually exclusive principal outcome domain
Forensic interpretative directness	High; moderate; low; no	Proximity and transferability of the study question to forensic toxicological interpretation or individual cause-of-death assessment

The use of this structure avoids combining evidentiary proximity with effect magnitude, statistical robustness, analytical validation, risk of bias, or certainty assessment.

2.3. Operational definition of forensic interpretative directness

Forensic interpretative directness asks how closely the population, exposure context, biological material, outcome, and interpretative objective of a study correspond to the use of molecular evidence

in postmortem toxicology or individual cause-of-death assessment. It does not ask whether the result was statistically significant or methodologically reliable.

Rating	Interpretation
High	The study directly examined human medicolegal, postmortem, fatal-toxicological, or cause-of-death evidence and explicitly related the molecular result to postmortem concentration interpretation, fatal opioid toxicity, differentiation of exposure from toxicity, or individual forensic causal attribution.
Moderate	The study examined severe toxicity, overdose, respiratory depression, fatal or perinatal outcomes, or a pharmacokinetic/pharmacodynamic mechanism with a clear and biologically plausible transfer to forensic interpretation, but did not directly validate use of the marker for individual postmortem cause-of-death attribution.
Low	The study primarily addressed therapeutic response, dose requirement, non-severe adverse effects, experimental pharmacokinetics, opioid use disorder, chronic exposure, or a non-specific molecular signature. Translation to fatal toxicity or postmortem interpretation required substantial extrapolation.
No	No meaningful transfer from the reported population, exposure, matrix, outcome, or molecular finding to opioid-related forensic toxicology or cause-of-death assessment could be established. Workbook values recorded as None were displayed as No.

A high-directness study may still have high risk of bias, inadequate precision, no independent validation, or very-low GRADE certainty. Conversely, a methodologically strong therapeutic pharmacogenetic study may have low forensic directness. The following attributes were therefore explicitly excluded from the directness rating: sample size, effect size, p value, confidence-interval width, multiple-testing correction, analytical validation, replication, risk of bias, and GRADE certainty.

T.D. and A.M. independently assigned the study-level directness ratings using the operational definitions, resolving disagreements through discussion to a final agreed classification. No pre-consensus agreement statistic is available; it cannot be reconstructed from final agreed ratings.

2.4. Distribution of directness

Forensic interpretative directness	Number of studies	Percentage
High	25	33.3%
Moderate	11	14.7%
Low	31	41.3%
No	8	10.7%
**Total**	**75**	**100.0%**

3. Main-text Figure 3 – Molecular-opioid co-occurrence in the included studies

3.1. Opioid assignment

Opioid categories were identified using deterministic, case-insensitive text matching of main opioid, all opioids investigated, and opioid class. Recognized categories were fentanyl/sufentanil, nitazenes, methadone, heroin, oxycodone, tramadol, morphine, codeine, and hydrocodone. A study was additionally assigned to mixed opioids when the extraction fields explicitly described mixed, multiple, polydrug, or unspecified multi-opioid exposure, or when more than one recognized opioid category was detected. An opioid outside the prespecified Figure 4 profile was retained as other/non-profiled opioid for the mapping audit and co-occurrence matrix rather than being forced into the mixed category.

This is a study-level co-occurrence mapping. In mixed-opioid studies, a link indicates that the opioid and molecular node occurred within the same study-level evidence record; it does not prove that the reported molecular association was specific to that opioid.

3.2. Molecular-node extraction

Molecular nodes comprised canonical gene symbols and predefined mechanism/omics categories. Gene symbols were identified using a fixed dictionary applied to the pharmacogenetic gene, variant, allele, and interpretation fields. The aliases MDR1 and NK1R were normalized to ABCB1 and TACR1, respectively. The fixed dictionary included candidate pharmacogenes, transporters, receptors, genome-wide loci, and signaling genes explicitly represented in the workbook. Variant identifiers, rs numbers, methylation-probe identifiers, chromosome labels, metabolites, and generic methodological terms were not treated as gene nodes.

Mechanism nodes were generated through predefined keyword rules:

Mechanism node	Principal detection terms
CYP-mediated metabolism	CYP, cytochrome, clearance, biotransformation
UGT glucuronidation	UGT, glucuronidation-related terms
Drug transport/efflux	ABCB1/MDR1, P-glycoprotein, drug transport, efflux
Opioid receptor signalling	OPRM1, OPRD1, OPRK1, μ-opioid or opioid-receptor terms
Catecholamine/pain modulation	COMT, catecholamine, dopamine, pain-modulation terms
Metabolomic signature	Explicit metabolomics terms; generic metabolism was not sufficient
Lipidomic alteration	Explicit lipidomics terms
Proteomic signature	Proteomics terms
Transcriptomic regulation	Transcriptomics, gene expression, mRNA, RNA-seq
Epigenetic regulation	Epigenetics, methylation, miRNA/microRNA, histone terms
Mitochondrial dysfunction	Mitochondrial, bioenergetic, oxidative, respiratory-chain terms
Neurotransmission/GPCR signalling	GABA, glutamate, GPCR, ERK/MAPK, neurotransmission terms
Inflammatory/immune signalling	Immune, inflammatory, cytokine terms

Genes and mechanisms detected within the same study were combined and duplicates were removed. Edge weight was then calculated as the number of unique studies supporting each opioid-node connection. For readability, only the 32 molecular nodes with the greatest total number of unique study-level links were displayed; ranking ties were resolved deterministically by node name. Matrix cell colour and printed counts represent the number of unique contributing studies. The matrix retains all 32 displayed nodes and all nonzero opioid-node counts from the original mapping. A blank cell indicates no mapped link in the included evidence; it is not proof of absence of a biological relationship.

4. Main-text Figure 4 – Descriptive forensic orientation, recency, and evidence coverage within the included literature

4.1. Analytical background

Quantitative profiles were calculated for nine opioid categories represented in the included evidence: fentanyl/sufentanil, mixed opioids, methadone, heroin, oxycodone, tramadol, morphine, codeine, and hydrocodone. Multi-label study-opioid attribution followed the deterministic rules described above.

Nitazenes had no contributing included study and were therefore retained only as a zero-evidence sentinel. No quantitative profile was assigned to nitazenes.

4.2. Forensic-event definition

For each opioid category i, the evidence volume n_i was the number of unique assigned studies. A study was classified as a forensic-event study when at least one of the following workbook fields was coded Yes: fatal outcome, postmortem study, cause of death outcome.

The number of forensic-event studies was denoted by f_i, and the within-opioid forensic-event proportion was:

$$q_i = \frac{f_i}{n_i}.$$

4.3. Forensic-setting representation component B_i

The absolute forensic-event count was normalized to the largest count observed among the nine profiled opioid categories:

$$f_i^* = \frac{f_i}{\max_j(f_j)}.$$

Forensic-setting representation was calculated as the geometric mean of normalized absolute forensic-event volume and within-opioid forensic-event proportion:

$$B_i = \sqrt{f_i^* q_i}.$$

This formulation combines the normalized count and within-category proportion of forensic-event studies. It limits domination by a large but predominantly non-forensic evidence base and prevents a very small category composed only of forensic reports from automatically receiving the maximum score. The component is a proxy for the representation of fatal, postmortem, or cause-of-death settings within this review corpus. It does not measure population incidence, mortality, prevalence, toxic potency, or health-service burden.

4.4. Multidimensional forensic-directness component D_i

The directness component used in Figure 4 is broader than the single fourth-axis variable displayed in Figure 2. Four study-level fields were used: directness to opioid toxicity, directness to fatal outcome, directness to forensic interpretation, and directness to postmortem toxicology.

The ordinal ratings were converted to numerical values (High → 1.000; Moderate → 0.667; Low → 0.333; None/No → 0.000).

For study s, multidimensional forensic directness was:

$$d_s = \frac{d_{\text{toxicity},s} + d_{\text{fatal},s} + d_{\text{forensic},s} + d_{\text{postmortem},s}}{4}.$$

For opioid category i, the component was the unweighted mean of the study-level scores assigned to that category:

$$D_i = \frac{1}{n_i}\sum_{s=1}^{n_i} d_s.$$

4.5. Recency component R_i

The median publication year of studies assigned to opioid $i$was denoted by m_i. The median, rather than the maximum year, was used to reduce sensitivity to a single recent publication. Median years were min-max normalized across the nine evidence-bearing categories:

$$R_i = \frac{m_i - \min_j(m_j)}{\max_j(m_j) - \min_j(m_j)}.$$

The resulting component ranges from 0 for the least recent opioid-specific evidence profile to 1 for the most recent profile. It describes the recency of the included evidence and is not an external epidemiological trend measure.

4.6. Forensic orientation and recency score F_i

The forensic orientation and recency score was the equally weighted arithmetic mean of forensic-setting representation, multidimensional forensic directness, and publication recency:

$$F_i = \frac{B_i + D_i + R_i}{3}.$$

Equal weighting was used to avoid fitting coefficients retrospectively to produce a preferred opioid ordering. The score describes the composition of the included literature and has no normative interpretation as a research, public-health, or casework priority. It is distinct from evidence support, which combines normalized evidence volume, molecular breadth, and the composite validation signal (Section 4.10).

4.7. Evidence-volume component V_i

Evidence volume was normalized to the largest observed number of contributing studies:

$$V_i = \frac{n_i}{\max_j(n_j)}.$$

The scale is zero-anchored: a hypothetical category with no evidence would score 0, while the category with the largest evidence volume scores 1.

4.8. Molecular-breadth component M_i

Pharmacogenetic breadth was the number of distinct canonical genes identified through the fixed dictionary across studies assigned to opioid i. Omics breadth was the number of distinct layers identified exclusively from the explicit omics-level field. Recognized layers were metabolomics, lipidomics, proteomics, transcriptomics, epigenetics, microbiomics, and integrated multi-omics. Generic references to drug metabolism were not interpreted as metabolomics.

Raw molecular breadth was:

$$L_i = g_i + o_i,$$

where g_i is the number of distinct pharmacogenetic genes and o_i is the number of distinct omics layers. It was normalized as:

$$M_i = \frac{L_i}{\max_j(L_j)}.$$

4.9. Composite validation signal C_i

Three study-level extraction fields were transformed into ordinal maturity scores: omics validation, integrated-model validation, and raw-omics-data availability. The transformations were predefined for visualization and are not validated measurement scales.

Component	Workbook category	Score
Omics validation	External or independent	1.00
Omics validation	Cross-validation, internal, biological, or technical validation	0.70
Omics validation	None	0.10
Omics validation	Not reported	0.05
Omics validation	Not applicable	0.00
Integrated-model validation	External or independent	1.00
Integrated-model validation	Cross-validation, internal, biological, or technical validation	0.70
Integrated-model validation	None or not applicable	0.00
Integrated-model validation	Not reported	0.05
Raw omics data	Yes	0.70
Raw omics data	Unclear	0.25
Raw omics data	No	0.10
Raw omics data	Not reported	0.05
Raw omics data	Not applicable	0.00

The small non-zero values for explicitly absent or unreported information preserve ordering between documented absence, non-reporting, and non-applicability without treating any of these categories as substantive validation. Raw-data availability was capped below 1 because availability facilitates reproducibility but does not itself constitute independent validation.

Study-level scores were averaged within each opioid category to obtain O_i, I_i, and A_i. The composite signal was:

$$C_i = 0.70O_i + 0.20I_i + 0.10A_i.$$

The greater weight assigned to omics validation reflects its direct relevance to molecular biomarker reproducibility; integrated-model validation and raw-data availability were treated as complementary signals. The index was used only for descriptive visualization.

4.10. Molecular evidence support S_i

Overall evidence support was the equal-weight mean of normalized evidence volume, normalized molecular breadth, and the composite validation signal:

$$S_i = \frac{V_i + M_i + C_i}{3}.$$

Fatal, postmortem, and cause-of-death fields were intentionally excluded from S_i, because they already contribute to B_i. This prevents the same information from being used on both sides of the gap calculation.

4.11. Evidence coverage gap G_i

The evidence coverage gap was defined as the non-negative difference between forensic orientation and recency and molecular evidence support:

$$G_i = \max(0, F_i - S_i).$$

A positive value indicates that the forensic orientation and recency of the included evidence exceed its relative volume, molecular breadth, and composite validation signal on the internal normalized scales. A zero value indicates only that evidence support equals or exceeds forensic orientation and recency; it does not establish evidence sufficiency.

4.12. Visual encoding

In Figure 4:

— horizontal position represents F_i, the forensic orientation and recency score;

— vertical position represents G_i, the evidence coverage gap;

— bubble area is proportional to n_i, the number of contributing studies, using area scaling rather than radius scaling, without weighting by participant numbers;

— bubble fill represents C_i, the composite validation signal;

— nitazenes are shown separately as a zero-evidence sentinel and are not positioned as a quantitative profile.

4.13. Generated forensic orientation and recency components

Opioid category	n_i	f_i	q_i	B_i	D_i	Median year	R_i	F_i
Heroin	15	12	0.800	0.661	0.694	2021	0.800	0.718
Mixed opioids	41	22	0.537	0.733	0.585	2020	0.700	0.673
Methadone	19	10	0.526	0.489	0.614	2020	0.700	0.601
Tramadol	23	10	0.435	0.445	0.601	2020	0.700	0.582
Fentanyl/sufentanil	15	4	0.267	0.220	0.483	2023	1.000	0.568
Hydrocodone	11	6	0.545	0.386	0.500	2021	0.800	0.562
Oxycodone	18	7	0.389	0.352	0.500	2021	0.800	0.551
Morphine	32	18	0.563	0.678	0.604	2015	0.200	0.494
Codeine	20	12	0.600	0.572	0.654	2013	0.000	0.409

4.14. Generated molecular evidence-support and coverage-gap components

Opioid category	PGx genes	Omics layers	Raw breadth	V_i	M_i	C_i	S_i	G_i
Heroin	14	5	19	0.366	0.380	0.403	0.383	0.336
Mixed opioids	45	5	50	1.000	1.000	0.240	0.747	0.000
Methadone	26	3	29	0.463	0.580	0.227	0.424	0.177
Tramadol	20	2	22	0.561	0.440	0.119	0.373	0.209
Fentanyl/sufentanil	34	3	37	0.366	0.740	0.232	0.446	0.122
Hydrocodone	20	2	22	0.268	0.440	0.273	0.327	0.235
Oxycodone	33	3	36	0.439	0.720	0.215	0.458	0.093

Opioid category	PGx genes	Omics layers	Raw breadth	V_i	M_i	C_i	S_i	G_i
Morphine	35	5	40	0.780	0.800	0.200	0.593	0.000
Codeine	24	3	27	0.488	0.540	0.164	0.397	0.011
Nitazenes	0	0	0	Not estimated	Not estimated	Not estimated	Not estimated	Not estimated

The tables report values to three decimal places; calculations in the analytical script retain full numerical precision.

5. Interpretative limits of the exploratory scores

Forensic orientation and recency, evidence support, the composite validation signal, and the evidence coverage gap are author-defined, internally normalized descriptive constructs. They describe the included 2008–March 2026 literature, not lethality, prevalence, or forensic risk. Their relative values depend on the eligible date window, available extraction fields, opioid assignment, selected coding rules, and fixed weights. Overlapping study membership makes opioid profiles dependent, and a single study can support several molecular nodes. No external calibration, empirical validation of the composite scales, uncertainty intervals, or review-level sensitivity analysis of the weights was performed. A zero gap means only that evidence support equals or exceeds forensic orientation and recency on these internal scales; it does not establish adequate evidence or clinical readiness. The scores must not be used to estimate individual risk, determine cause of death, or rank epidemiological or public-health priorities.
