# Supplementary material for: Pharmacogenetic Evidence in Opioid-Related Toxicity and Death with an Appraisal of Emerging Multi-Omics Studies: A Systematic Review
Source: Int J Mol Sci. 2026 Sep 19;27(18):8332. doi: 10.3390/ijms27188332 (PMC13607749; doi:10.3390/ijms27188332)
Supplement: Supplementary file 1 [file ijms-27-08332-s001.zip › TableS1_Search_strategies.pdf]

Supplementary Table S1. Complete database search strategies and eligibility limits.

**MEDLINE (PubMed)**
("analgesics, opioid"[mh] OR "opioid-related disorders"[mh] OR opioid*[tiab] OR opiate*[tiab] OR heroin[tiab] OR morphine[tiab] OR methadone[tiab] OR buprenorphine[tiab] OR oxycodone[tiab] OR tramadol[tiab] OR codeine[tiab] OR fentanyl[tiab] OR carfentanil[tiab] OR acetylfentanyl[tiab] OR furanylfentanyl[tiab] OR acrylfentanyl[tiab] OR nitazene[tiab] OR isotonitazene[tiab] OR "U-47700"[tiab] OR "U-50488"[tiab] OR "AH-7921"[tiab] OR "MT-45"[tiab] OR benzimidazole[tiab] OR hydrocodone[tiab] OR hydromorphone[tiab]) AND ("pharmacogenetics"[mh] OR pharmacogen*[tiab] OR polymorphism*[tiab] OR "genetic variant*"[tiab] OR biomarker*[tiab] OR metabolomic*[tiab] OR proteomic*[tiab] OR lipidomic*[tiab] OR transcriptomic*[tiab] OR "multi*omic*"[tiab] OR epigenetic*[tiab] OR methylation[tiab] OR microRNA[tiab] OR miRNA [tiab] OR lncNRA[tiab] OR microbiom*[tiab]) AND ("toxicology"[mh] OR forensic[tiab] OR toxic*[tiab] OR postmortem[tiab] OR "post-mortem"[tiab] OR "cause*of death"[tiab] OR overdose*[tiab] OR intoxication*[tiab])
**Scopus**
TITLE-ABS-KEY ((opioid* OR opiate* OR heroin OR morphine OR methadone OR buprenorphine OR oxycodone OR tramadol OR codeine OR fentanyl OR carfentanil OR acetylfentanyl OR furanylfentanyl OR acrylfentanyl OR nitazene OR isotonitazene OR "U-47700" OR "U-50488" OR "AH-7921" OR "MT-45" OR benzimidazole OR hydrocodone OR hydromorphone) AND (pharmacogen* OR polymorphism* OR "genetic variant*" OR biomarker* OR metabolomic* OR proteomic* OR lipidomic* OR transcriptomic* OR multiomic* OR "multi-omic*" OR epigenetic OR methylation* OR microRNA* OR miRNA* OR lncRNA* OR microbiom*) AND (forensic OR toxic* OR postmortem OR "post-mortem" OR "cause of death" OR "causes of death" OR overdose* OR intoxication*))
**Web of Science Core Collection**
(opioid* OR opiate* OR heroin OR morphine OR methadone OR buprenorphine OR oxycodone OR tramadol OR codeine OR fentanyl OR carfentanil OR acetylfentanyl OR furanylfentanyl OR acetylfentanyl OR nitazene OR isotonitazene OR "U-47700" OR "U-50488" OR "AH-7921" OR "MT-45" OR benzimidazole OR hydrocodone OR hydromorphone) (Topic) and (pharmacogen* OR polymorphism* OR "genetic variant*" OR biomarker* OR metabolomic* OR proteomic* OR lipidomic* OR transcriptomic* OR multiomic* OR "multi-omic*" OR epigenetic OR methylation* OR microRNA* OR miRNA* OR lncRNA* OR microbiom*) (Topic) and (forensic OR toxic* OR postmortem OR "post-mortem" OR "cause of death" OR "causes of death" OR overdose* OR intoxication*) (Topic)

Note. Formal-review eligibility was restricted to English-language, peer-reviewed original studies published from 1 January 2008; the main database search was completed in March 2026. The strings above reproduce the recorded strategies; their publication-date and language limits are specified here and in Section 2.2. A separate historical search on 14 September 2026 applied the same strings to publications before 1 January 2008 in the same three databases. It did not change the formal 75-study corpus. Procedures, yields, and the retained historical reports are documented in Supplementary Table S5.

Nitazene search terms and retrieval. The explicit terms were nitazene, isotonitazene, and benzimidazole, used as PubMed title/abstract terms, Scopus TITLE-ABS-KEY terms, and Web of Science Topic terms in the strings above. A focused check of the archived title/abstract screening register identified at least 10 records naming nitazenes or named derivatives in their titles. All were recorded as excluded before full-text assessment; none appears among the 112 full texts. Supplementary Table S4 reproduces their bibliographic identifiers and original exclusion reasons. This minimum title-

based count is not a complete count of all records containing a nitazene term anywhere in their metadata and does not imply an absence of nitazene research.

Historical coverage. The supplementary search identified 414 records (MEDLINE/PubMed 52; Scopus 294; Web of Science Core Collection 68). After removal of 112 duplicates, 302 records were screened, 292 were excluded at title/abstract, and all ten reports assessed in full text were retained. Ten is a strategy-specific minimum under the PEOS interpretation detailed in Supplementary Table S5. Sindrup 1990 and Kirchheiner 2007 were not returned and remain separate contextual references. The mandatory toxicity/forensic block and incomplete molecular terminology limit sensitivity for otherwise relevant metabolism/response studies. The recorded PubMed string contains lncNRA[tiab], a misspelling of lncRNA; it is reproduced verbatim to avoid implying that a corrected search was performed.
