# Supplementary material for: Pharmacogenetic Evidence in Opioid-Related Toxicity and Death with an Appraisal of Emerging Multi-Omics Studies: A Systematic Review
Source: Int J Mol Sci. 2026 Sep 19;27(18):8332. doi: 10.3390/ijms27188332 (PMC13607749; doi:10.3390/ijms27188332)
Supplement: Supplementary file 1 [file ijms-27-08332-s001.zip › TableS2_Complete_study_characteristics.pdf]

Supplementary Table S2. Principal characteristics of the 75 studies included in the review.

Study, country and year	Study design	Analyzed sample (n)	Opioid	Exposure context	Molecular domain	Principal targets	Biological matrix	Analytical approach	Primary outcome	Validation	RoB
Naruge et al., Japan (2020) **[45]**	Single-arm quasi-experimental	34; PGx subgroup 25	Tramadol/ acetaminophen	Therapeutic treatment of chemotherapy-induced neuropathy	Pharmacogenetics	CYP2D6	Peripheral blood	xTAG CYP2D6 Luminex assay	Analgesic response and treatment-limiting toxicity	None	Moderate
Frost et al., Norway (2012) **[46]**	Forensic case series	34	Codeine	Fatal intoxication	Pharmacogenetics and toxicology	CYP2D6	Femoral blood	Allele-specific and long-range PCR; postmortem toxicology	Morphine formation, metabolic ratios, and cause-of-death interpretation	None	Low
St Sauver et al., USA (2017) **[47]**	Cohort	219	Codeine, tramadol, oxycodone, hydrocodone	Therapeutic exposure	Clinical pharmacogenetics	CYP2D6	Peripheral blood and electronic health records	Luminex genotyping and copy-number analysis	Poor pain control or opioid-related adverse reactions	None	Moderate
Elkalioubie et al., France (2011) **[48]**	Case report	1	Tramadol	Intentional near-fatal overdose	Pharmacogenetics and pharmacokinetics	CYP2D6, CYP2B6	Serial plasma samples	Exon sequencing, TaqMan copy-number analysis, LC-MS/MS	Refractory cardiotoxicity and multiorgan toxicity	None	Low
Enaba et al., Egypt (2015) **[49]**	Analytical cross-sectional	74	Tramadol	Misuse or dependence	Candidate-gene pharmacogenetics	OPRM1, ABCB1	Peripheral blood and urine	PCR-RFLP and toxicological screening	Tramadol-associated seizures	None	Moderate
Wong et al., Australia (2024) **[50]**	Cohort	54	Multiple opioids, predominantly oxycodone	Therapeutic cancer-pain treatment	Pharmacogenetics	COMT, OPRM1	Peripheral blood	Genome-array genotyping	Opioid dose, pain severity, and adverse-effect phenotype	None	Moderate
Thumtecho et al., Thailand (2023) **[51]**	Case report	1	Tramadol	Therapeutic exposure	Pharmacogenetics and phenoconversion	CYP2D6	Peripheral blood	Clinical genotyping and toxicological screening	Depersonalization/derealization syndrome	None	Low
Annis et al., USA (2025) **[52]**	Biobank cohort	39,519; opioid-naive subset 33,450	Multiple prescription opioids	Postoperative therapeutic exposure	Candidate-locus genomic association	OPRM1 and other opioid-related loci	Genomic DNA and linked EHR data	Genotyping array and prescription-data analysis	Persistent opioid use after surgery	Internal replication analyses	Moderate
Corradin et al., USA (2022) **[53]**	Postmortem case-control	102	Mixed opioids	Fatal overdose	Epigenomics and chromatin profiling	H3K27ac regulatory regions	DLPFC neuronal nuclei	FANS, ChIP-seq, and machine learning	Molecular classification of fatal opioid overdose	Repeated internal cross-validation	Low
Rompala et al., USA (2023) **[54]**	Postmortem case-control	38	Mixed opioids	Opioid use disorder	Neuronal methylomics and hydroxymethylomics	Genome-wide 5mC and 5hmC loci	Orbitofrontal-cortex neuronal nuclei	Genome-wide methylation and hydroxymethylation profiling	Neuronal epigenetic dysregulation in OUD	Internal sensitivity and external-reference comparison	Moderate
Ahmadimanesh et al., Iran (2023) **[55]**	Analytical cross-sectional	44	Tramadol	Poisoning and fatal intoxication	Pharmacogenetics	CYP2D6	Blood and cerebrospinal fluid	Sanger sequencing and HPLC	Tramadol and metabolite concentrations by CYP2D6 phenotype	None	Low
Aly et al., France/Egypt (2022) **[56]**	Postmortem case series	48	Tramadol	Fatal intoxication	Pharmacogenetics and toxicology	CYP2D6	Postmortem blood	Nested PCR, Sanger sequencing, copy-number analysis, LC-MS/MS	Metabolic-ratio discrimination of CYP2D6 phenotype	None	Low

Study, country and year	Study design	Analyzed sample (n)	Opioid	Exposure context	Molecular domain	Principal targets	Biological matrix	Analytical approach	Primary outcome	Validation	RoB
Mendez et al., USA (2023) **[57]**	Translational quasi-experimental	Two iPSC donors; postmortem comparison 20	Morphine	Experimental exposure and postmortem OUD	Transcriptomics and epigenomics	Morphine-responsive transcriptional and epigenetic pathways	iPSC-derived cortical neurons and DLPFC	RNA sequencing and methylation profiling	Concordance between experimental morphine responses and postmortem OUD	Biological and cross-dataset validation	Low
Grimm et al., USA (2023) **[58]**	Postmortem case-control	Brain RNA 42; blood miRNA 26	Mixed opioids	Opioid use disorder	Transcriptomics and miRNA	Brain-blood miRNA-mRNA networks	DLPFC and peripheral blood	RNA-seq and miRNA-seq	Cross-tissue regulatory-network dysregulation	Independent tissue-level comparison	Moderate
Chighine et al., Italy (2022) **[59]**	Metabolomic case report with historical comparators	Total 27	Methadone	Fatal infant intoxication	Metabolomics	Urinary metabolic profile	Postmortem urine	Proton NMR and confirmatory toxicology	Fatal-intoxication and terminal metabolic phenotype	None	Moderate
Aly et al., France/Egypt (2025) **[60]**	Postmortem case series	48	Tramadol	Fatal and non-fatality-classified postmortem cases	Multigene pharmacogenetics	UGT1A8, UGT2B7, ABCC2, SLC22A1	Postmortem blood	HaloPlex targeted NGS and LC-MS/MS	Genetic determinants of tramadol metabolic ratios	None	Low
Chanfreau-Coffinier et al., USA (2022) **[61]**	National retrospective cohort	2,436,654	Codeine, hydrocodone, tramadol	Therapeutic prescribing	Drug-drug-gene interaction analysis	CYP2D6	Genomic and prescription databases	Database linkage and pharmacogenetic phenotype assignment	Prevalence of actionable drug-gene and drug-drug-gene interactions	None	Moderate
Ozberk et al., Australia (2022) **[62]**	Cohort	46	Methadone	Therapeutic cancer-pain treatment	Pharmacogenetics	ARRB2	Peripheral blood	TaqMan genotyping with Sanger confirmation	Methadone requirements and pain severity	Technical genotype validation	Moderate
Shu et al., China (2024) **[63]**	Multicenter RCT	573	Sufentanil	Epidural labor analgesia	Genotype-guided pharmacogenetics	CYP3A4	Peripheral blood	Bidirectional sequencing and TaqMan PCR	Analgesic equivalence and maternal adverse effects	None	Moderate
Aly et al., France/Egypt (2023) **[64]**	Postmortem case series	48	Tramadol	Postmortem intoxication assessment	Multigene pharmacogenetics	CYP2D6, CYP2B6, CYP3A4	Postmortem blood	HaloPlex targeted NGS and LC-MS/MS	Genotype-metabolic-ratio associations and fatality classification	None	Low
Sullivan et al., USA (2025) **[65]**	Integrated secondary multi-omic analysis	Source-cohort size not uniform across datasets	Heroin, fentanyl, hydrocodone, oxycodone, mixed opioids	OUD and overdose	Integrated multi-omics	Convergent neurobiological gene networks	Predominantly postmortem brain datasets	GRIN network analysis and random-walk modelling	Identification of convergent opioid-addiction genes and druggable networks	Five-fold cross-validation and internal network filtering	Moderate
Russell et al., USA (2023) **[66]**	Case report	1	Hydrocodone and polypharmacy	Therapeutic exposure	Multigene clinical pharmacogenetics	Drug-metabolizing enzymes and transporters	Buccal swab	Commercial CLIA-certified PGx panel	Medication-related oversedation, fall, and prescribing cascade	None	Low
Lambert et al., USA (2024) **[67]**	Analytical cross-sectional	821	Multiple opioids, with contemporary fentanyl predominance	Non-fatal overdose	Candidate-gene pharmacogenetics	CYP3A5, DRD2, NK1R	Buccal DNA	Targeted 180-SNP panel	Lifetime non-fatal opioid overdose	None	Moderate
Radhakrishna et al., USA (2025) **[68]**	Analytical cross-sectional	96	Prenatal mixed-opioid exposure	Neonatal opioid withdrawal syndrome	Placental epigenomics	Pain-regulatory genes, including OPRM1	Placenta	Illumina MethylationEPIC array	NOWS requiring pharmacological treatment	None	Low
Sürmen et al., Turkey (2026) **[69]**	Postmortem case-control	48	Heroin	Chronic illicit use and OUD	Proteomics	Region-specific neural proteins	Hippocampus, putamen, caudate	LC-ESI-MS/MS	Heroin-associated regional protein dysregulation	None	Low

Study, country and year	Study design	Analyzed sample (n)	Opioid	Exposure context	Molecular domain	Principal targets	Biological matrix	Analytical approach	Primary outcome	Validation	RoB
Elmsjö et al., Sweden (2022) **[70]**	Postmortem case-control	934	Oxycodone	Fatal intoxication	Untargeted metabolomics	Acylcarnitines and related metabolites	Postmortem peripheral blood	LC-HRMS and OPLS-DA	Discrimination of oxycodone intoxication from other deaths	External validation	Moderate
Grahl et al., USA (2025) **[71]**	Analytical cross-sectional	115	Multiple hospital-administered opioids	Naloxone-treated acute toxicity	Multigene clinical pharmacogenetics	Thirteen-gene PGx panel	Buccal DNA	PharmacoScan	Pharmacogenetic profiles in patients requiring naloxone	None	Moderate
Radhakrishna et al., USA (2024) **[72]**	Analytical cross-sectional	96	Prenatal mixed-opioid exposure	OUD and NOWS	Placental methylomics	SLC, ABC, and PKC pathways	Placenta	Illumina MethylationEPIC array	Transporter methylation and NOWS classification	None	Low
Minnai et al., multicenter Europe (2025) **[73]**	Genome-wide analytical	2,052	Morphine, fentanyl, oxycodone, buprenorphine	Therapeutic cancer-pain treatment	Genome-wide pharmacogenomics	Genome-wide variants	Genomic DNA	Axiom Precision Medicine array and imputation	Opioid-associated nausea and vomiting	None	Low
Drevin et al., France (2021) **[74]**	Forensic case report	1	Heroin with venlafaxine, paroxetine, and ethanol	Fatal mixed intoxication	Pharmacogenetics and phenoconversion	CYP2D6, CYP2C19	Femoral blood	TaqMan genotyping and LC-MS/MS	Explanation of discordant drug metabolism in a fatality	None	Moderate
Ettienne et al., USA (2019) **[75]**	Cohort	113	Buprenorphine/ naloxone	OUD treatment	Clinical pharmacogenetics	CYP3A4, CYP3A5	Clinical genotype records and urine	Clinical PGx testing and urine toxicology	Withdrawal under pharmacogenetically guided versus standard dosing	None	High
Wendt et al., Finland/USA (2020) **[76]**	Postmortem genome-wide association	Discovery 136; replication 99	Tramadol	Postmortem detection	Genome-wide pharmacogenomics	Genome-wide variants affecting tramadol metabolism	Postmortem blood	Omni2.5Exome array and replication sequencing	Genetic determinants of tramadol metabolic ratios	Independent replication cohort	Moderate
Wang et al., China (2020) **[77]**	Analytical cross-sectional	74	Heroin with or without HIV infection	Chronic illicit exposure	Exosomal miRNA	Circulating miRNA panel	Plasma exosomes	miRNA array and RT-PCR confirmation	Heroin- and HIV-associated circulating miRNA profiles	Technical RT-PCR validation	Moderate
Amante et al., Italy (2021) **[78]**	Analytical cross-sectional	177	Fentanyl	Therapeutic and analytical exposure classification	Untargeted metabolomics	Urinary metabolic signature	Urine	Reversed-phase and HILIC LC-HRMS with SIMCA	Detection of fentanyl-associated metabolic profiles	Ten-fold cross-validation	Moderate
Iwersen-Bergmann et al., Germany/Austria (2021) **[79]**	Postmortem analytical	107	Methadone	Fatal intoxication	Pharmacogenetics and tissue distribution	ABCB1	Medulla oblongata and femoral blood	PCR-based mass-spectrometric genotyping and GC-MS	Medulla-to-blood methadone distribution	None	Low
Ahmad et al., USA (2017) **[80]**	Postmortem case-control	380	Methadone	Fatal intoxication	Pharmacogenetics	CYP2B6	Postmortem blood	TaqMan genotyping and methadone toxicology	Allele enrichment and methadone concentration	None	Moderate
Talal et al., USA (2020) **[81]**	Analytical cross-sectional	97	Methadone	Stable OUD treatment	Pharmacogenetics and pharmacokinetics	CYP2B6	Plasma	Agena iPLEX and enantioselective LC-MS/MS	R- and S-EDDP/methadone ratios	None	Low
Shu et al., USA (2021) **[82]**	Postmortem case-control	153	Mixed opioids	Fatal intoxication	Epigenome-wide methylation	Genome-wide CpGs and epigenetic age	Postmortem DLPFC	Illumina MethylationEPIC array	Acute opioid-intoxication methylation signature	None	Moderate
Chou and Hsu, Taiwan (2021) **[83]**	Cohort	111	Morphine	Postoperative analgesia	Pharmacogenetics	OPRM1	Genomic DNA	PCR and capillary sequencing	PCA morphine consumption by genotype and sex	None	Moderate

Study, country and year	Study design	Analyzed sample (n)	Opioid	Exposure context	Molecular domain	Principal targets	Biological matrix	Analytical approach	Primary outcome	Validation	RoB
Arafa and Atteia, Egypt (2018) **[84]**	Cohort	120	Tramadol	Long-term therapeutic exposure	Pharmacogenetics and biochemical toxicology	CYP2D6	Blood and urine	Luminex CYP2D6 assay and biochemical measurements	Oxidative injury and hepatotoxicity	None	Moderate
Grimsrud et al., USA (2019) **[85]**	Pharmacokinetic pilot	13	Fentanyl	Therapeutic administration in burn patients	Pharmacogenetics and pharmacokinetics	CYP2D6, CYP3A4	Plasma	Targeted genotyping, LC-MS/MS, and population PK modelling	Fentanyl clearance and early plasma exposure	None	Low
Liu et al., USA (2021) **[86]**	Postmortem analytical	Expression 40; methylation 30; paired 29	Mixed opioids	Opioid use disorder	Integrated transcriptomics and methylomics	Cross-omic regulatory modules	DLPFC	RNA sequencing and genome-wide methylation analysis	Integrated OUD-associated regulatory modules	None	Low
Yu et al., Republic of Korea (2018) **[87]**	Analytical pharmacokinetic	23	Tramadol	Controlled administration	Pharmacogenetics	CYP2D6	Hair	Pyrosequencing, long-range PCR, and column-switching LC-MS/MS	Hair tramadol-metabolite gradients by genotype	Technical assay validation	Low
Egervari et al., USA/Hungary (2017) **[88]**	Translational postmortem	Sample size varied by molecular experiment	Heroin	Fatal and chronic exposure	Chromatin and transcriptomic multi-omics	H3K27ac, GRIA1, glutamatergic pathways	Dorsal striatum	Microarray, ChIP, ATAC-seq, and animal experiments	Chromatin dysregulation and translational modulation	Human-animal biological validation	Low
Loh et al., USA (2021) **[89]**	Cohort	107	Multiple CYP2D6-metabolized opioids	Chronic-pain treatment	Clinical pharmacogenetics	CYP2D6	Clinical genotype data	Next-generation sequencing	Pain scores and medication changes after PGx testing	None	High
Wendt et al., Finland/USA (2019) **[90]**	Postmortem analytical	208	Tramadol	Postmortem detection	Pathway-based multigene pharmacogenetics	CYP2D6, UGT2B7, and additional pathway genes	Postmortem blood	Targeted MPS, LC-MS/MS, and machine learning	Tramadol metabolizer-phenotype classification	Cross-validation	Low
Wendt et al., Finland/USA (2019) **[91]**	Postmortem analytical	CYP2D6 sequencing 44	Tramadol	Postmortem detection	Full-gene pharmacogenetics and machine learning	CYP2D6	Postmortem blood	Long-range PCR, MPS, and supervised classification	Genotype and metabolizer-phenotype prediction	Cross-validation	Low
Muriel et al., Spain (2019) **[92]**	Longitudinal cohort	Baseline 88; completers 83	Multiple prescription opioids	Chronic pain with prescription OUD	Multigene pharmacogenetics	OPRM1, COMT, ABCB1, OPRD1, ARRB2	Saliva	TaqMan PCR	Adverse-event burden during opioid tapering	None	Moderate
Jakobsson et al., Sweden (2021) **[93]**	Postmortem analytical	174	Oxycodone	Intoxication and non-intoxication deaths	Pharmacogenetics and toxicology	CYP2D6	Femoral blood	Pyrosequencing, ddPCR, and LC-MS/MS	Oxymorphone/oxycodone ratio and cause-of-death discrimination	None	Low
Rowsell et al., Australia (2019) **[94]**	Double-blind crossover RCT	60	Morphine	Experimental administration in obstructive sleep apnea	Pharmacogenetics and respiratory pharmacodynamics	OPRM1, ABCB1, HTR3B	Plasma and genomic DNA	Targeted genotyping and LC-MS/MS	Sleep-related hypoxemia and ventilatory response	None	Moderate
Wu and Kearney, USA (2013) **[95]**	Case report	1	Codeine	Therapeutic use before a motor-vehicle accident	Multigene pharmacogenetics and phenoconversion	CYP2D6, UGT2B7	Peripheral blood	Mass-spectrometric PGx panel and LC-MS/MS	Toxicological interpretation of driving impairment	None	Moderate

Study, country and year	Study design	Analyzed sample (n)	Opioid	Exposure context	Molecular domain	Principal targets	Biological matrix	Analytical approach	Primary outcome	Validation	RoB
Samer et al., Switzerland (2010) **[96]**	Crossover pharmacology RCT	10	Oxycodone	Controlled experimental administration	Pharmacogenetics and drug-interaction pharmacology	CYP2D6	Plasma	AmpliChip CYP450 and LC-MS/MS	Analgesia, respiratory effects, and adverse reactions	None	Low
Madadi et al., Canada/USA (2011) **[97]**	Multigenerational family case report	Index fatal infant and maternal family	Codeine	Breastfeeding-associated fatal neonatal toxicity	Pharmacogenetics and copy-number inheritance	CYP2D6	Maternal and familial blood; infant postmortem specimens	CYP2D6 genotyping and copy-number analysis	Familial transmission of ultrarapid metabolism	None	High
Daws et al., USA/Sweden/Hungary (2013) **[98]**	Postmortem case-control	47	Heroin	Chronic illicit exposure	Transcriptomics, protein signalling, and pharmacogenetics	ELK1, OPRM1	Striatal brain regions	Microarray, Western blotting, immunoassays, and TaqMan genotyping	Dysregulation of the ELK1-μ-opioid-receptor network	Technical molecular validation	Moderate
Kharasch et al., USA (2015) **[99]**	Controlled pharmacokinetic	64	Methadone	Experimental administration	Pharmacogenetics and pharmacokinetics	CYP2B6	Plasma	Fluidigm/TaqMan genotyping and stereoselective LC-MS/MS	Methadone concentrations, clearance, and metabolism	None	Low
Andresen et al., Germany (2013) **[100]**	Forensic case series	9 successfully genotyped	Codeine/morphine and mixed toxicological cases	Postmortem detection	Forensic toxicogenetics	CYP2D6, CYP2C19	Postmortem blood	AmpliChip, pyrosequencing, and GC-MS	Explanation of anomalous parent/metabolite ratios	None	Moderate
Fonseca et al., Portugal (2016) **[101]**	Postmortem case series	97	Tramadol	Postmortem detection	Forensic pharmacogenetics	CYP2D6	Postmortem blood	Sanger sequencing and GC-MS	Identification of poor metabolizers from metabolic ratios	Technical sequencing validation	Low
Lötsch et al., Germany (2009) **[102]**	Controlled pharmacology	57	Codeine	Experimental administration	Pharmacogenetics and phenotyping	CYP2D6	Plasma and urine	Pyrosequencing, dextromethorphan phenotyping, and LC-MS/MS	Prediction of extreme morphine formation	Cross-validation	Moderate
Kolesnikov et al., Estonia (2011) **[103]**	Cohort	102	Morphine	Postoperative PCA	Pharmacogenetics	OPRM1, COMT	Genomic DNA	PCR, sequencing, and restriction analysis	Morphine requirement and central adverse effects	None	Low
Christoffersen et al., Denmark (2016) **[104]**	Postmortem case-control	977	Morphine and methadone	Fatal opioid-addiction cases	Multigene forensic pharmacogenetics	Twenty-nine candidate SNPs	Postmortem blood	TaqMan OpenArray and LC-MS/MS	Genotype frequencies in deceased versus living opioid-dependent subjects	None	Moderate
Jannetto and Bratanow, USA (2009) **[105]**	Cohort	61	Oxycodone, tramadol, methadone, hydrocodone	Chronic-pain treatment	Pharmacogenetics and therapeutic drug monitoring	CYP2D6	Plasma	Pyrosequencing and LC-MS/MS	Steady-state concentrations, analgesia, and adverse reactions	None	Moderate
Bunten et al., UK (2010) **[106]**	Postmortem case series	40	Methadone	Fatal intoxication	Pharmacogenetics	CYP2B6, OPRM1	Postmortem blood	PCR/Sanger or TaqMan genotyping and HPLC	Methadone concentration and co-intoxicant distribution	None	Moderate
Oertel et al., Germany (2012) **[107]**	Case-control translational	73	Heroin	Chronic illicit exposure	Integrated pharmacogenetics and epigenetics	OPRM1	Postmortem brain and genomic DNA	Pyrosequencing, expression, receptor-density, and signalling assays	OPRM1 genotype-methylation-expression interaction	Independent cohort	Moderate

Study, country and year	Study design	Analyzed sample (n)	Opioid	Exposure context	Molecular domain	Principal targets	Biological matrix	Analytical approach	Primary outcome	Validation	RoB
Madadi et al., Canada (2010) **[108]**	Fatal pediatric case report	1	Hydrocodone	Therapeutic exposure with interacting medications	Pharmacogenetics and drug-drug-gene interactions	CYP2D6 and CYP3A pathways	Postmortem blood	TaqMan genotyping and GC-MS	Fatal hydrocodone toxicity with absent hydromorphone	None	Low
Kesimci et al., Turkey (2012) **[109]**	Cohort	83	Fentanyl	Spinal anesthesia	Pharmacogenetics	ABCB1	Genomic DNA	PCR-RFLP	Respiratory depression, oxygenation, and sedation	None	Low
Pogliani et al., Italy (2012) **[110]**	Neonatal case report	1	Morphine	Treatment of neonatal abstinence syndrome	Pharmacogenetics	ABCB1, UGT2B7, OPRM1, COMT	Blood and urine	Pyrosequencing	Morphine-associated urinary retention and acute kidney injury	None	Low
Ferreirós et al., Germany (2009) **[111]**	Pediatric twin case report	2	Codeine	Severe and fatal therapeutic intoxication	Pharmacogenetics and pharmacokinetics	CYP2D6	Blood, CSF, urine, and brain	PCR, copy-number analysis, LC-MS/MS, and PK simulation	Explanation of divergent toxicity in twins	None	Low
Madadi et al., Canada (2009) **[112]**	Case-control	72	Codeine	Maternal use during breastfeeding	Multigene pharmacogenetics	CYP2D6, UGT2B7	Maternal blood/plasma, breast milk, and infant postmortem data	SNaPshot and PCR-RFLP	Infant CNS depression and maternal toxicity	None	Low
Lam et al., Canada (2014) **[113]**	Postmortem case-control	68	Codeine	Fatal intoxication	Multigene forensic pharmacogenetics and phenoconversion	CYP2D6, ABCB1, UGT2B7, OPRM1, COMT	Femoral blood	SNaPshot, TaqMan genotyping, and LC-MS/MS	Codeine/morphine concentrations and metabolic ratios	None	Moderate
Richards-Waugh et al., USA (2014) **[114]**	Postmortem case-control	228	Methadone	Fatal intoxication	Forensic pharmacogenetics	CYP3A4	Postmortem blood spots	TaqMan allelic discrimination	Variant enrichment and methadone concentration	None	Moderate
Bastami et al., Sweden (2014) **[115]**	Cohort	40	Morphine	Postoperative PCA	Multigene pharmacogenetics	UGT2B7, OPRM1, ABCB1	Plasma and genomic DNA	Pyrosequencing and HPLC	Morphine dose and glucuronide formation	None	Low
Sistonen et al., Canada/Netherlands (2012) **[116]**	Case-control	111	Codeine	Maternal use during breastfeeding	Multilocus pharmacogenetics	CYP2D6, ABCB1, UGT2B7, OPRM1, COMT	Maternal genomic DNA	SNaPshot and TaqMan assays	Maternal and infant CNS depression	None	Low
Bunten et al., UK (2011) **[117]**	Postmortem case series	40	Methadone	Fatal intoxication	Pharmacogenetics	CYP2B6, OPRM1	Postmortem blood	TaqMan genotyping and GC-MS	Methadone and benzodiazepine concentrations	None	Moderate
Shaw et al., Canada (2012) **[118]**	Clinical case report	1	Codeine	Suspected overdose versus analgesic failure	Pharmacogenetics	CYP2D6	Clinical blood sample	CYP2D6 genotyping	Resolution of an ambiguous opioid adverse-event presentation	None	Low
Stamer et al., Germany (2008) **[119]**	Case report	1	Tramadol	Postoperative therapeutic exposure	Pharmacogenetics and pharmacokinetics	CYP2D6	Serial plasma samples	PCR, copy-number analysis, and enantioselective LC-MS/MS	Delayed respiratory depression	None	Low

Abbreviations: ABC, ATP-binding cassette; CLIA, Clinical Laboratory Improvement Amendments; CNV, copy-number variation; CSF, cerebrospinal fluid; CV, cross-validation; DDGI, drug-drug-gene interaction; DGI, drug-gene interaction; DLPFC, dorsolateral prefrontal cortex; EHR, electronic health record; FANS, fluorescence-activated nuclei sorting; GC-MS, gas chromatography-mass spectrometry; HPLC, high-performance liquid chromatography; LC-HRMS, liquid chromatography-high-resolution mass spectrometry; LC-MS/MS, liquid chromatography-tandem mass spectrometry; MPS, massively parallel sequencing; NGS, next-generation sequencing; NMR, nuclear magnetic resonance; NOWS, neonatal opioid withdrawal syndrome; OPLS-DA, orthogonal partial least-squares discriminant analysis; OUD, opioid use disorder; PCA, patient-controlled analgesia; PGx, pharmacogenetics/pharmacogenomics; PK, pharmacokinetics; RoB, risk of bias.
