# Supplementary material for: Pharmacogenetic Evidence in Opioid-Related Toxicity and Death with an Appraisal of Emerging Multi-Omics Studies: A Systematic Review
Source: Int J Mol Sci. 2026 Sep 19;27(18):8332. doi: 10.3390/ijms27188332 (PMC13607749; doi:10.3390/ijms27188332)
Supplement: Supplementary file 1 [file ijms-27-08332-s001.zip › TableS3_GRADE_evidence_profile.pdf]

Supplementary Table S3. Four outcome-focused GRADE evidence profiles.

Population: opioid-exposed humans, living or deceased. Comparisons: reference genotype, phenotype, exposure group or molecular profile, as applicable. Studies and endpoints are heterogeneous; no pooled effect estimate was calculated. The original evidence-map counts are retained and should not be interpreted as homogeneous outcome-specific sample sizes.

1. Pharmacogenetic associations with opioid toxicity or death

51 studies in the primary PGx category. The category includes indirect PK and therapeutic endpoints; 51 is the evidence-map denominator, not 51 studies of a single toxicity outcome.

Summary of findings. The most coherent findings concern CYP2D6-dependent codeine/tramadol bioactivation, CYP2B6-dependent methadone disposition, and selected ABCB1-related distribution effects. Association magnitude and precision differ by gene, opioid, population and endpoint. The evidence does not provide one pooled effect or support deterministic attribution of an individual death.

GRADE domain	Judgment and rationale
Risk of bias	Serious. Recurrent limitations concern exposure reconstruction, co-medications, polysubstance use, ancestry, phenoconversion and outcome definition; selected genotype groups are small or case based.
Inconsistency	Serious. Comparable gene–opioid associations vary across populations and phenotype definitions, with positive and null findings and limited direct replication. Different opioids and different endpoints are not assumed to estimate one common effect.
Indirectness	Serious. A substantial portion evaluates PK, metabolic ratios, analgesia or non-fatal adverse effects, requiring extrapolation to severe toxicity, fatal outcomes and individual postmortem attribution.
Imprecision	Serious. Rare genotype subgroups and case-based evidence produce wide confidence intervals or incompletely reported uncertainty; no common pooled estimate was calculated.
Publication bias	Uncertain; no additional downgrade. Non-publication of negative evidence could not be quantified. Small exploratory cohorts, feature selection, and limited independent validation warrant caution but do not establish an excess of unpublished negative studies. Language and dissemination restrictions remain review-level limitations.
Upgrading	No upgrading factor applied. Large estimates in selected studies, biological plausibility, or high internal AUCs did not establish a robust, replicated body-level effect, dose-response relationship, or residual confounding necessarily attenuating the association.

Certainty: VERY LOW. Observational association evidence starts at low certainty; the serious limitations above reduce confidence to very low. Ratings have a floor of very low and are not arithmetic sums of downgrades below that floor. Importance: critical.

2. Molecular discrimination of opioid exposure and adverse outcomes

15 non-integrated omics studies: 11 transcriptomic/epigenetic, 3 metabolomic and 1 proteomic. The 2 primarily integrated investigations are discussed separately in Part B. Counts describe contributing domains, not a common classifier or diagnostic target.

Summary of findings. Studies report exposure-associated molecular alterations and selected internally evaluated discrimination. Replication of signatures across independent populations, tissues, platforms and laboratories is limited. Exposure or OUD discrimination is not equivalent to discrimination of opioid-specific fatal causation; the single proteomic observation supplies no validated diagnostic performance.

GRADE domain	Judgment and rationale
Risk of bias	Serious. Small discovery cohorts and high-dimensional feature selection, heterogeneous preprocessing, variable multiple-testing control, and frequent reliance on internal evaluation create substantial risk of overoptimistic estimates.

GRADE domain	Judgment and rationale
Inconsistency	Serious. Candidate features and molecular signals are incompletely reproduced across studies. Performance estimates from different targets, populations and matrices are not treated as interchangeable or pooled.
Indirectness	Serious. Many signatures characterize exposure, chronic use or OUD, rather than severe toxicity or death attributable specifically to an opioid.
Imprecision	Serious. Discovery and validation samples are generally limited relative to model complexity, with incomplete uncertainty estimates and uncertain stability of selected features.
Publication bias	Uncertain; no additional downgrade. Non-publication of negative evidence could not be quantified. Small exploratory cohorts, feature selection, and limited independent validation warrant caution but do not establish an excess of unpublished negative studies. Language and dissemination restrictions remain review-level limitations.
Upgrading	No upgrading factor applied. Large estimates in selected studies, biological plausibility, or high internal AUCs did not establish a robust, replicated body-level effect, dose-response relationship, or residual confounding necessarily attenuating the association.

Certainty: VERY LOW. Observational association evidence starts at low certainty; the serious limitations above reduce confidence to very low. Ratings have a floor of very low and are not arithmetic sums of downgrades below that floor. Importance: critical.

3 Molecular attribution of opioid toxicity and death in forensic settings

The 75-study corpus was examined for evidence relevant to this question. Only subsets directly address forensic or postmortem endpoints; 75 is not a count of diagnostic-accuracy studies. Study-level directness categories are descriptive and were not converted into GRADE ratings.

Summary of findings. Selected PGx and postmortem findings can inform explanations of unusual parent-drug/metabolite patterns and tissue distribution. The corpus does not provide precise, independently established diagnostic performance for individual cause-of-death attribution. This profile concerns the evidence for molecular associations and discrimination in forensic settings; the separate claim of incremental utility is appraised narratively.

GRADE domain	Judgment and rationale
Risk of bias	Serious. Relevant limitations include retrospective exposure reconstruction, co-intoxicant assessment, incompletely specified outcomes, and incomplete clinical or postmortem information.
Inconsistency	Not assessable for a common forensic effect; no additional downgrade. The studies address different molecular findings and attribution questions, and do not permit a common forensic effect estimate. Variation in directness categories alone is not statistical inconsistency.
Indirectness	Serious. Much of the evidence addresses clinical, PK or exposure outcomes rather than validated discrimination or attribution of an individual opioid-related death.
Imprecision	Serious. The directly informative subsets are small and heterogeneous, with no precise, independently validated estimates for individual attribution.
Publication bias	Uncertain; no additional downgrade. Non-publication of negative evidence could not be quantified. Small exploratory cohorts, feature selection, and limited independent validation warrant caution but do not establish an excess of unpublished negative studies. Language and dissemination restrictions remain review-level limitations.
Upgrading	No upgrading factor applied. Large estimates in selected studies, biological plausibility, or high internal AUCs did not establish a robust, replicated body-level effect, dose-response relationship, or residual confounding necessarily attenuating the association.

Certainty: VERY LOW. Observational association evidence starts at low certainty; the serious limitations above reduce confidence to very low. Ratings have a floor of very low and are not arithmetic sums of downgrades below that floor. Importance: critical.

4 Reproducibility and transportability of molecular findings

The 75-study corpus was assessed for evidence that molecular associations or discriminative performance reproduce across independent settings. There is no single common replication endpoint. This makes explicit the reproducibility component of the original synthesis-level question; it is not a global certainty grade for the review.

Summary of findings. Independent evaluation is limited and heterogeneous. Six of the 43 studies in the quantitative subset received the original external/independent validation code. This encompasses independent cohorts, cross-dataset or cross-tissue comparisons, and agreement with external reference data; it does not mean six independently validated forensic classifiers. These categories remain descriptive, without being translated mechanically into certainty levels.

GRADE domain	Judgment and rationale
Risk of bias	Serious. Retrospective or discovery-based analyses, selected samples, residual confounding and incompletely independent evaluation limit confidence in reproducibility.
Inconsistency	Not assessable for a common replicated effect; no additional downgrade. Different genes, signatures, tissues and endpoints cannot by themselves establish inconsistency of one association.
Indirectness	Serious. Cross-tissue, mechanistic or reference-data agreement does not directly establish transportable performance for individual opioid toxicity or cause-of-death assessment.
Imprecision	Serious. Independent tests are few and often small, and uncertainty for external performance is incompletely reported.
Publication bias	Uncertain; no additional downgrade. Non-publication of negative evidence could not be quantified. Small exploratory cohorts, feature selection, and limited independent validation warrant caution but do not establish an excess of unpublished negative studies. Language and dissemination restrictions remain review-level limitations.
Upgrading	No upgrading factor applied. Large estimates in selected studies, biological plausibility, or high internal AUCs did not establish a robust, replicated body-level effect, dose-response relationship, or residual confounding necessarily attenuating the association.

Certainty: VERY LOW. Observational association evidence starts at low certainty; the serious limitations above reduce confidence to very low. Ratings have a floor of very low and are not arithmetic sums of downgrades below that floor. Importance: critical.

Illustrative independent-evaluation material is reported in Chighine et al. **[59]**, Oertel et al. **[107]**, Elmsjö et al. **[70]**, Mendez et al. **[57]**, Rompala et al. **[54]** and Grimm et al. **[58]**. Citation numbers refer to the revised manuscript bibliography. The descriptive EV code does not imply the same validation design across these reports.

5. Two structured narrative appraisals

These questions concern incremental utility and study methodology. They are retained in the review but are not assigned GRADE certainty levels. This distinction was introduced in response to peer review; it does not change study eligibility or the 75-study corpus.

Incremental interpretative utility of molecular integration

Evidence considered. Two studies were primarily classified as integrated PGx/omics investigations. They explore different molecular layers and endpoints and support possible biological convergence between inherited susceptibility and downstream regulation.

What was demonstrated. The observations can inform mechanistic hypotheses and the conceptual framework. Neither study directly tests the incremental benefit of an integrated assessment over conventional scene investigation, autopsy, histopathology and toxicology for resolving borderline concentrations or attributing an individual death.

Appraisal. Incremental forensic utility remains untested. No effect size for added utility, diagnostic improvement, or reduction in attribution error can be estimated from this evidence. No GRADE rating is assigned to the utility claim.

Identification and control of confounding

Evidence considered. Reporting across all 75 studies was examined for polysubstance exposure, co-medications, comorbidities, ancestry, tolerance, renal/hepatic function, phenoconversion, clinical context, analytical factors, agonal conditions and postmortem handling.

What was demonstrated. Adjustment, restriction, matching and sensitivity analyses were used in selected studies, but the factors measured and their reporting varied. Many case-based and exploratory investigations remain vulnerable to incomplete exposure reconstruction and residual confounding.

Appraisal. Confounder control is a methodological property, not a patient or forensic outcome. Relevant limitations inform risk-of-bias or indirectness judgments for the outcome under consideration; they do not constitute a separate GRADE-rated outcome.

Methodological notes

GRADE is applied to outcome-focused bodies, separately from author-defined directness and validation categories. The appraisal does not treat biological plausibility, number of features, or a large internal AUC as a validated forensic benefit. Non-publication and selective reporting could not be quantified; the unsupported "strongly suspected" publication-bias designation was removed and no additional downgrade was made solely on that basis. Multiplicity-adjusted null findings are present in the included corpus **[82]**.

Methodological sources: GRADE guidance cited in the revised manuscript **[42-44]**, including Guyatt et al., GRADE guidelines 2 (doi: 10.1016/j.jclinepi.2010.09.012) and GRADE guidelines 5 (doi: 10.1016/j.jclinepi.2011.01.011).
