# Supplementary material for: Pharmacogenetic Evidence in Opioid-Related Toxicity and Death with an Appraisal of Emerging Multi-Omics Studies: A Systematic Review
Source: Int J Mol Sci. 2026 Sep 19;27(18):8332. doi: 10.3390/ijms27188332 (PMC13607749; doi:10.3390/ijms27188332)
Supplement: Supplementary file 1 [file ijms-27-08332-s001.zip › TableS4_Nitazene_screening_records.pdf]

Supplementary Table S4. Nitazene records and recorded title and abstract exclusion reasons.

This table documents retrieval of at least 10 nitazene-related reports in the original search. It reproduces the existing screening decisions and categorical reason labels; it does not introduce new exclusion decisions or alter the 75 included studies.

Report title	Year	DOI	Recorded exclusion reason
In silico metabolite prediction and LC-HRMS confirmation for forensic analysis of a fatal case involving novel synthetic opioid N, N-dimethyl etonitazene	2025	10.1093/jat/bkaf099	wrong outcome
In vitro, in vivo metabolism and quantification of the novel synthetic opioid N-piperidinyl etonitazene (etonitazepipne)	2024	10.1515/cclm-2023-1360	wrong outcome
In Vitro Metabolism of a Benzofuran-Substituted Nitazene: Ethyleneoxynitazene	2025	10.3390/metabo15100679	wrong population
Human metabolism of four synthetic benzimidazole opioids: isotonitazene, metonitazene, etodesnitazene, and metodesnitazene	2024	10.1007/s00204-024-03735-0	wrong outcome
A Comparative In Vitro Metabolic Study of Butonitazene Using Camel Liver and Fungus Cunninghamella elegans for Doping Control Applications	2026	10.1002/rcm.70005	wrong outcome; wrong population
Clinical and biochemical profile of a patient with acute metonitazene intoxication	2024	10.1016/j.jns.2024.122942	wrong outcome
Rapid screening of 2-benzylbenzimidazole nitazene analogs in suspect counterfeit tablets using Raman, SERS, DART-TD-MS, and FT-IR	2023	10.1002/dta.3440	wrong study design
Metabolic characterization of the new benzimidazole synthetic opioids - nitazenes	2024	10.3389/fphar.2024.1434573	wrong outcome; wrong population
Human metabolism and pharmacological profiling of protonitazepyne and metonitazepyne, two highly potent nitazenes: prediction of main metabolite activity based on μ-opioid receptor docking simulations	2026	10.1007/s00204-025-04163-4	wrong outcome
Isotonitazene Quantitation and Metabolite Discovery in Authentic Forensic Casework	2021	10.1093/JAT/BKAA016	wrong outcome

Screening status: all ten records were marked “Excluded” at title/abstract screening. Recorded combinations were wrong outcome (6), wrong outcome plus wrong population (2), wrong population (1), and wrong study design (1). Multiple reason labels can therefore apply to one record.

Records were located using nitazen, nitazep or nitazyn in the title fields of the original title/abstract-screening RIS export (1771 records). All ten were recorded as excluded at that stage and none was matched by DOI, normalized title or Rayyan identifier in the full-text-screening export (112 records). Reasons are reproduced from the original Rayyan export; multiple labels may apply to one record. This documentary audit establishes at least ten retrieved nitazene-related records and does not provide an exhaustive search of nitazene literature or a reassessment of eligibility.

Source snapshots: Rayyan title/abstract export dated 11 April 2026 (1771 records) and full-text export dated 26 June 2026 (112 records). Bibliographic titles, years and DOI identifiers are transcribed from the archived records. The absence of these 10 records from the full-text file does not exclude incidental mention of a nitazene in another report.
