# Supplementary material for: Pharmacogenetic Evidence in Opioid-Related Toxicity and Death with an Appraisal of Emerging Multi-Omics Studies: A Systematic Review
Source: Int J Mol Sci. 2026 Sep 19;27(18):8332. doi: 10.3390/ijms27188332 (PMC13607749; doi:10.3390/ijms27188332)
Supplement: Supplementary file 1 [file ijms-27-08332-s001.zip › TableS5_Pre2008_audit.pdf]

Supplementary Table S5. Supplementary pre-2008 eligibility audit and historical-reference inventory.

Purpose, procedure, and counting rule

On 14 September 2026, the original MEDLINE/PubMed, Scopus, and Web of Science Core Collection strategies were applied to publications before 1 January 2008, retaining their search terms and three-block concept structure (Supplementary Table S1). After deduplication, T.D. and A.M. independently screened titles/abstracts and assessed full texts using the formal review's selection procedures, resolving disagreements through discussion. The 10 retained reports were assessed against the non-temporal PEOS criteria, with the human-tissue interpretation specified for Bhasker in note A. They were not added to the formal 2008-March 2026 synthesis.

Audit component	Documented count / disposition
Records identified before deduplication	414: MEDLINE/PubMed 52; Scopus 294; Web of Science Core Collection 68
Duplicate records removed	112
Records screened by title/abstract	302
Records excluded at title/abstract screening	292
Reports sought for retrieval / not retrieved	10 / 0
Reports assessed in full text / excluded in full text	10 / 0
Reports retained in the historical inventory	10: 9 with in vivo exposure; 1 with in vitro exposure of human liver microsomes
Previously cited reports not returned by the strategies	Sindrup 1990 and Kirchheiner 2007 (Part B); outside the 10-report search-yield count
Formal review corpus	75 studies; historical reports not added and analytical outputs not recalculated

Note. This is a strategy-specific minimum under the stated PEOS interpretation.

Database-retrieved reports

All reports below were retained in the supplementary historical inventory. All remain outside the formal synthesis because they were published before 1 January 2008. “Retained” does not denote inclusion in the 75-study corpus.

Report and bibliographic identification	P and E: population / opioid exposure	O: original molecular analysis and source location	S, audit disposition, and qualification
**1. Bhasker (2000) [120]**<br>Bhasker CR, McKinnon W, Stone A, et al. Genetic polymorphism of UDP-glucuronosyltransferase 2B7 (UGT2B7) at amino acid 268: ethnic diversity of alleles and potential clinical significance. Pharmacogenetics 2000;10(8):679-85. doi: 10.1097/00008571-200011000-00002.	**P:** 28 human liver donors genotyped; morphine activity measured in microsomes from 26 livers. Additional human DNA samples informed allele-frequency analyses.<br>**E:** Morphine used directly as a substrate in donor-derived liver microsomes. Exposure is in vitro; donor exposure in vivo is not documented.	**PGx:** UGT2B7 His268Tyr (*1/*2) characterized and compared with morphine 3-glucuronidation activity in human microsomes. The null intergenotypic comparison remains an eligible analysis.<br>**Source:** Samples, UGT activity methods, and Results, pp. 680-683.	**S:** Original functional experimental study.<br>**Audit:** Retained under the human-biospecimen/experimental interpretation of PEOS.<br>**Qualification:** Not a clinical morphine-exposure study.
**2. Jannetto (2002) [121]**<br>Jannetto PJ, Wong SH, Gock SB, et al. Pharmacogenomics as molecular autopsy for postmortem forensic toxicology: genotyping cytochrome P450 2D6 for oxycodone cases. J Anal Toxicol 2002;26(7):438-47. doi: 10.1093/jat/26.7.438.	**P:** 15 human autopsy cases; 26 volunteers provide a genotype comparison group.<br>**E:** Oxycodone documented by toxicological investigation and represented in the selected death certifications.	**PGx:** Direct CYP2D6 *3/*4/*5 analysis integrated with oxycodone concentrations, co-exposures, and case interpretation. The analysis is original despite non-significant overall genotype-frequency differences.<br>**Source:** Methods, pp. 439-441; Tables I-III.	**S:** Retrospective autopsy investigation with a control genotype group.<br>**Audit:** Retained.<br>**Qualification:** Genotyping does not itself establish the cause of death.
**3. Lötsch (2002) [122]**<br>Lötsch J, Zimmermann M, Darimont J, et al. Does the A118G polymorphism at the μ-opioid receptor gene protect against morphine-6-glucuronide toxicity? Anesthesiology 2002;97(4):814-9. doi: 10.1097/00000542-200210000-00011.	**P:** 2 men with severe renal dysfunction.<br>**E:** Both received oral morphine; morphine and morphine-6-glucuronide concentrations were measured in relation to different clinical tolerability.	**PGx:** OPRM1 variants, including A118G, and transporter variants in MDR1/ABCB1 and MRP2/ABCC2 were examined by PCR/sequencing and related to morphine/M6G response.<br>**Source:** Genetic methods, p. 816; Results and Table 3, p. 817.	**S:** Original two-case clinical report.<br>**Audit:** Retained.<br>**Qualification:** The proposed protective effect is exploratory and is not a validated individual-risk estimate.
**4. Levo (2003) [123]**<br>Levo A, Koski A, Ojanperä I, Vuori E, Sajantila A. Post-mortem SNP analysis of CYP2D6 gene reveals correlation between genotype and opioid drug (tramadol) metabolite ratios in blood. Forensic Sci Int 2003;135(1):9-15. doi: 10.1016/S0379-0738(03)00159-2.	**P:** 33 Finnish human autopsy cases.<br>**E:** Selection required tramadol positivity in postmortem blood; tramadol and O-/N-demethylated metabolites were measured.	**PGx:** CYP2D6 SNPs and gene deletion/amplification were determined. Functional-allele groups were compared with parent-drug/metabolite ratios.<br>**Source:** Sections 2.1-2.4, pp. 10-12; Results, p. 13; Table 2.	**S:** Original observational postmortem study.<br>**Audit:** Retained.<br>**Qualification:** Tramadol detection does not mean all 33 deaths were caused by tramadol.

Report and bibliographic identification	P and E: population / opioid exposure	O: original molecular analysis and source location	S, audit disposition, and qualification
**5. Gasche (2004) [34]**<br>Gasche Y, Daali Y, Fathi M, et al. Codeine intoxication associated with ultrarapid CYP2D6 metabolism. N Engl J Med 2004;351(27):2827-31. doi: 10.1056/NEJMoa041888.	**P:** A 62-year-old patient with a severe clinical intoxication.<br>**E:** Oral codeine, 25 mg three times daily, given for cough; codeine, morphine, and glucuronides were measured.	**PGx:** Leukocyte-DNA analysis documented CYP2D6 duplication/multiduplication; metabolic phenotyping and drug concentrations informed the interpretation.<br>**Source:** Methods, p. 2828; genotype/phenotype Results, pp. 2829-2830.	**S:** Original case report published as a Brief Report.<br>**Audit:** Retained; 1 of the 10, not an extra contextual record.<br>**Qualification:** Interacting drugs and renal impairment also contributed to the proposed explanation.
**6. Klepstad (2004) [124]**<br>Klepstad P, Rakvåg TT, Kaasa S, et al. The 118 A > G polymorphism in the human μ-opioid receptor gene may increase morphine requirements in patients with pain caused by malignant disease. Acta Anaesthesiol Scand 2004;48(10):1232-9. doi: 10.1111/j.1399-6576.2004.00517.x.	**P:** 207 cancer-pain patients genotyped; 99 with adequately controlled pain entered the principal pharmacological comparison.<br>**E:** Documented oral morphine treatment.	**PGx:** Four OPRM1 polymorphisms were directly assayed; genotypes were compared with morphine dose requirements and drug/metabolite concentrations.<br>**Source:** Pharmacogenetic analyses, pp. 1233-1234; Tables 3 and 7.	**S:** Original clinical observational study.<br>**Audit:** Retained for opioid response.<br>**Qualification:** Overdose or fatality is not required by the non-temporal outcome criterion.
**7. Jin (2005) [125]**<br>Jin M, Gock SB, Jannetto PJ, Jentzen JM, Wong SH. Pharmacogenomics as molecular autopsy for forensic toxicology: genotyping cytochrome P450 3A4*1B and 3A5*3 for 25 fentanyl cases. J Anal Toxicol 2005;29(7):590-8. doi: 10.1093/jat/29.7.590.	**P:** 25 human autopsy cases.<br>**E:** Fentanyl detected in toxicological investigation, including fentanyl-toxicity and mixed-drug cases.	**PGx:** CYP3A4*1B and CYP3A5*3 genotyped by pyrosequencing and compared with fentanyl/norfentanyl findings.<br>**Source:** Methods, pp. 591-592; Results and Table I, pp. 592-595.	**S:** Original postmortem observational investigation.<br>**Audit:** Retained.<br>**Qualification:** Non-significant comparisons and case-level causal uncertainty limit interpretation, not PEOS correspondence.
**8. Drakenberg (2006) [126]**<br>Drakenberg K, Nikoshkov A, Horváth MC, et al. μ Opioid receptor A118G polymorphism in association with striatal opioid neuropeptide gene expression in heroin abusers. Proc Natl Acad Sci USA 2006;103(20):7883-8. doi: 10.1073/pnas.0600871103.	**P:** Human postmortem brain material; 118 subjects genotyped overall (initial 65 plus 53 additional subjects); molecular/peptide measurements used a separate subset of 53 from the initial 65.<br>**E:** Heroin exposure supported by toxicology and case history.	**PGx:** OPRM1 A118G directly genotyped and related to heroin-associated striatal transcript/peptide differences.<br>**Source:** Genotype-stratified Results, pp. 7884-7886; Methods, pp. 7887-7888.	**S:** Original postmortem association study.<br>**Audit:** Retained as PGx, not independent multi-omics.<br>**Qualification:** Targeted mRNA/peptide assays; baseline genotype effects cannot be fully separated from exposure effects.

Report and bibliographic identification	P and E: population / opioid exposure	O: original molecular analysis and source location	S, audit disposition, and qualification
**9. Koren (2006) [127]**<br>Koren G, Cairns J, Chitayat D, Gaedigk A, Leeder SJ. Pharmacogenetics of morphine poisoning in a breastfed neonate of a codeine-prescribed mother. Lancet 2006;368(9536):704. doi: 10.1016/S0140-6736(06)69255-6.	**P:** Mother-infant clinical/autopsy investigation.<br>**E:** Maternal codeine prescription and reported morphine measurements in milk and infant blood. Milk-mediated fatal causation is the authors' interpretation.	**PGx:** CYP2D6 genotype analysis identified maternal gene duplication and characterized family/infant genotypes. The infant was not reported to carry the maternal ultrarapid genotype.<br>**Source:** Original Case Report, p. 704.	**S:** Original case report.<br>**Audit:** Retained for PEOS correspondence only.<br>**Integrity flag:** Expression of Concern (2026); see note C. Not treated as established causal evidence.
**10. Lee (2006) [128]**<br>Lee HK, Lewis LD, Tsongalis GJ, et al. Negative urine opioid screening caused by rifampin-mediated induction of oxycodone hepatic metabolism. Clin Chim Acta 2006;367(1-2):196-200. doi: 10.1016/j.cca.2005.11.030.	**P:** A 60-year-old patient receiving chronic-pain treatment.<br>**E:** Oxycodone therapy and urinary oxycodone metabolites documented during rifampin co-treatment.	**PGx:** CYP2D6, CYP3A4, and CYP3A5 genotyped and integrated with the metabolite profile to assess the explanation for negative oxycodone screens.<br>**Source:** Section 3.2 and Table 3, p. 198; Discussion, p. 199.	**S:** Original case report.<br>**Audit:** Retained.<br>**Qualification:** A predominant drug-drug interaction explanation does not erase the actual opioid-specific PGx analysis.

PGx, pharmacogenetics/pharmacogenomics; M6G, morphine-6-glucuronide; SNP, single-nucleotide polymorphism. Source locations refer to printed article pages and section/table labels in the reviewed full texts.

Notes on eligibility, interpretation, and integrity

A. Human-derived material and exposure. Bhasker is retained under an explicit interpretation that the PEOS human-biospecimen and experimental-study provisions cover opioid exposure of donor-derived material in vitro. The protocol does not expressly distinguish in vitro from in vivo exposure. Morphine administration to the donors was not documented. The inventory therefore comprises 9 reports with documented in vivo exposure plus 1 human-microsome experiment; the 10-report total depends on this stated interpretation and is not presented as 10 in vivo clinical/forensic studies.

B. PGx is not interchangeable with multi-omics. Every report has a directly investigated genetic component linked to an opioid. Targeted measurements of drug metabolites, individual transcripts, or peptides do not independently establish an omics study. Drakenberg is retained for genotype-associated biological findings in heroin-exposed human tissue, not for a claimed integrated multi-omics platform. Neither a null association nor an exploratory result is, by itself, an outcome-based exclusion.

C. Koren integrity notice. An Expression of Concern was published online on 3 February 2026 **[129]**. The report is counted here solely to document the outcome of the non-temporal PEOS audit; its inclusion in an inventory does not establish the validity of the proposed milk-mediated fatal mechanism. No evidence estimate from this historical case is added to the formal synthesis.

D. What this audit does not show. The count does not establish historical completeness, validate the sensitivity of the original strategy, or determine whether adding all earlier or otherwise missed studies would change the review's conclusions. The formal analytical outputs remain unchanged because these reports were not added, not because a sensitivity reanalysis proved that they would make no difference.
